# Projected Number of People With Onchocerciasis–Loiasis Coinfection in Africa, 1995 to 2025

**DOI:** 10.1093/cid/ciz647

**Published:** 2019-07-13

**Authors:** Natalie V S Vinkeles Melchers, Luc E Coffeng, Michel Boussinesq, Belén Pedrique, Sébastien D S Pion, Afework H Tekle, Honorat G M Zouré, Samuel Wanji, Jan H Remme, Wilma A Stolk

**Affiliations:** 1 Department of Public Health, Erasmus MC, University Medical Center Rotterdam, The Netherlands; 2 Unité Mixte Internationale 233 TransVIHMI, Institut de Recherche pour le Développement (IRD), INSERM U1175, University of Montpellier, Montpellier, France; 3 Research & Development Department, Drugs for Neglected Diseases initiative, and, Geneva, Switzerland; 4 Preventive Chemotherapy and Transmission Control Unit, Control of Neglected Tropical Diseases Department, World Health Organization, Geneva, Switzerland; 5 Expanded Special Project for Elimination of Neglected Tropical Diseases (ESPEN), World Health Organization, Regional Office for Africa, Cité du Djoué, Brazzaville, Republic of Congo; 6 Parasites and Vectors Research Unit, Department of Microbiology and Parasitology, University of Buea, Cameroon; 7 Ornex, France

**Keywords:** *Loa loa*, onchocerciasis, ONCHOSIM, mass drug administration, serious adverse events

## Abstract

**Background:**

Onchocerciasis elimination through mass drug administration (MDA) is hampered by coendemicity of Loa loa, as people with high L. loa microfilariae (mf) density can develop serious adverse events (SAEs) after ivermectin treatment. We assessed the geographical overlap of onchocerciasis and loiasis prevalence and estimated the number of coinfected individuals at risk of post-ivermectin SAEs in West and Central Africa from 1995 to 2025.

**Methods:**

Focusing on regions with suspected loiasis transmission in 14 countries, we overlaid precontrol maps of loiasis and onchocerciasis prevalence to calculate precontrol prevalence of coinfection by 5 km2 × 5 km2 pixel, distinguishing different categories of L. loa mf intensity. Using statistical and mathematical models, we predicted prevalence of both infections and coinfection for 2015 and 2025, accounting for the impact of MDA with ivermectin.

**Results:**

The number of people infected with onchocerciasis was predicted to decline from almost 19 million in 1995 to 4 million in 2025. Of these, 137 000 people were estimated to also have L. loa hypermicrofilaremia (≥20 000 L. loa mf/mL) in 1995, declining to 31 000 in 2025. In 2025, 92.8% of coinfected cases with loiasis hypermicrofilaremia are predicted to live in hypoendemic areas currently not targeted for MDA.

**Conclusions:**

Loiasis coinfection is a major concern for onchocerciasis elimination in Africa. We predict that under current strategies, at least 31 000 coinfected people still require treatment for onchocerciasis in 2025 while being at risk of SAEs, justifying continued efforts in research and development for safer drugs and control strategies.

## Teaser

***Onchocerciasis elimination using*** mass drug administration with ivermectin is jeopardized in areas with *Loa loa* coendemicity. ***W***e ***estimate the number of people at risk of*** serious adverse events ***due to ivermectin in onchocerciasis– loiasis coendemic areas in Africa between 1995 and 2025.***

In 2012 the World Health Organization targeted onchocerciasis, also known as river blindness, for elimination through preventive chemotherapy interventions. Onchocerciasis is caused by the filarial nematode *Onchocerca volvulus* and is transmitted by black flies (genus *Simulium*). Onchocerciasis has a long history of control. The Onchocerciasis Control Programme (1974–2002) in West Africa started with regional vector control, later supplemented by mass drug administration (MDA) with ivermectin. The African Programme for Onchocerciasis Control (APOC, 1995–2015) subsequently initiated MDA in 19 additional African countries. In 2016, about 132 million people at risk for *O. volvulus* infection in Africa were treated with ivermectin [1].

The elimination of onchocerciasis is hampered by coendemicity of loiasis (African eye worm), another filarial infection present in West and Central Africa. People with high *Loa loa* microfilarial (mf) densities can develop potentially fatal serious adverse events (SAEs) after ivermectin treatment. The pathogenesis of postivermectin *L. loa*–related neurological complications is not fully understood but is most likely a combination of mechanical blockage of capillaries by large numbers of *L. loa* mf paralyzed by the drug and vascular endothelial changes associated with the destruction of mf [2, 3]. Ivermectin can induce marked adverse effects with a functional impairment that lasts several days in those harboring >8000 mf/mL blood and unconsciousness, coma, and death in individuals with >30 000 mf/mL levels prior to treatment [2, 4, 5]. The probability of SAEs after ivermectin intake increases from approximately 0.7% in individuals with 30 000 mf/mL to approximately 7% in individuals with *L. loa* counts of 50 000 mf/mL [3]. As the benefits of treatment were considered to outweigh the risk of SAEs, coendemic areas with high onchocerciasis prevalence were nevertheless targeted for MDA with ivermectin, although fear for SAEs sometimes resulted in low treatment coverage [6]. However, coendemic areas with low onchocerciasis prevalence have remained untreated to date and might be in need of alternative strategies.

To understand the need for and feasibility of alternative strategies for onchocerciasis elimination in loiasis-endemic areas, we assessed the extent of coendemicity and estimated the number of coinfected individuals, including people with such high *L. loa* mf intensity levels that ivermectin treatment is considered unsafe. We combined empirical data using multiple sources with statistical and mathematical models to estimate how the number of onchocerciasis, loiasis, and coinfection cases change over time since precontrol levels up to 2025.

## METHODS

### General Approach

To estimate the number of people with onchocerciasis–loiasis coinfection in Africa, we first overlaid 5 km^2^ × 5 km^2^ resolution raster precontrol maps of loiasis and onchocerciasis prevalence with rural population density data from 1995. For each raster cell, we then used predictive modeling approaches to assess likely changes in the prevalence of both infections thanks to locally implemented MDA with ivermectin. Predictions for 2015 and 2025 were combined with raster maps of estimated population density for the same years, and results were subsequently aggregated over all raster cells within defined target areas. Below, we describe the methods used to estimate the impact of MDA with ivermectin on the 2 infections. A detailed methodology description, including the definitions applied, is provided in Supplement S1, Table S1.

### Geographical Scope of the Analysis

The geographical scope of this analysis includes areas that were surveyed for *L. loa* endemicity across APOC countries using the rapid assessment procedure for loiasis (RAPLOA) procedure [7, 8]. RAPLOA surveyed areas across countries and APOC projects that were suspected to be endemic of loiasis. See Supplement S1, Figure S1 for RAPLOA-surveyed areas that are coendemic for onchocerciasis.

### Data

#### Loiasis Maps

**Figure 1.**
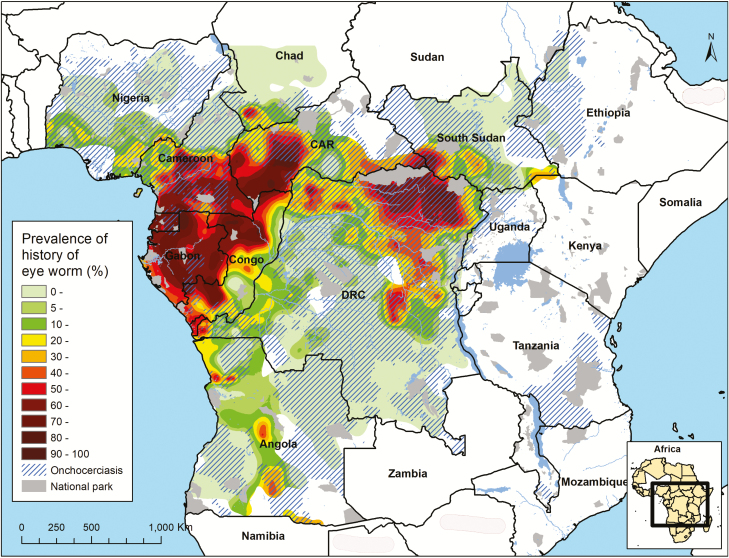
Map of the estimated precontrol overlap between the prevalence of palpable onchocercal nodules and the prevalence of a history of eye worm in African Programme for Onchocerciasis Control countries. Abbreviations: CAR, Central African Republic; DRC, Democratic Republic of Congo.

We used loiasis prevalence maps based on RAPLOA surveys that were performed by APOC to identify areas where SAEs might occur [4, 9, 10]. A loiasis prevalence raster map was previously generated by geostatistical kriging analysis of the RAPLOA data for all potentially endemic areas in 14 countries [11]. We updated this map to capture new RAPLOA data from Nigeria and Angola (Supplement S1, Table S2). The updated map is shown with onchocerciasis overlap in Figure 1, and a loiasis-only version is included in Supplement S1, Figure S3, and section 3.2. Next, we used the prevalence of history of eye worm from the RAPLOA map to estimate the proportion of people in each *L. loa* mf count intensity category (≥8000 to <20 000 mf/mL; ≥20 000 to <30 000 mf/mL; ≥30 000 mf/mL) using a statistical model as described in Supplement S1, section 3.4, and Figure S4.

#### Onchocerciasis Maps

We used previously published maps based on rapid epidemiological mapping of onchocerciasis (REMO) [12, 13]. A previous model-based geostatistical analysis of REMO data [12] resulted in a raster map of the precontrol prevalence of nodules in APOC countries (Figure 1) and some newly added extensions (Figure S2 and Supplement S1section 3.3 in). We converted the precontrol nodule prevalence map into a 5 km^2^ × 5 km^2^ map of precontrol *O. volvulus* mf prevalence in the general population using methods described elsewhere [14, 15].

We linked each raster cell to its APOC project (implementation unit for MDA, see definitions in Supplement S1, section 1) and related information on project treatment history and expected future treatment scenario. Briefly, nearly all onchocerciasis hyper- and mesoendemic areas had started annual or sometimes biannual MDA by 2013 (up to which year we have reported treatment history). For the few projects that had not yet started MDA by 2013, we followed the assumptions of Kim et al [16], with some adaptations described in Supplement S1, section 3.5, and Supplement S3.

### Impact of MDA on Loiasis

Based on published studies [17–19], we assume that MDA causes a change in the *L. loa* mf count frequency distribution. In our main analysis, we assumed that MDA causes a change in the *L. loa* mf count frequency distribution among the population that received a first ivermectin treatment according to a Markov transition matrix derived from Gardon et al [17] (Table 1). We assumed that the *L. loa* mf count frequency distribution was sustained after subsequent treatment without any further changes. After applying the transition matrix per pixel, linear interpolation was carried out to categorize the data into the required *L. loa* mf intensity classes: mf-negative; >0 to <8000; ≥8000 to <20 000; ≥20 000 to <30 000; ≥30 000 mf/mL (see Supplement S1, section 4.1). We generated raster maps of the predicted prevalence of *L. loa* hypermicrofilaremia (≥20 000 mf/mL) for 1995, 2015, and 2025. The cutoff of *L. loa* mf loads ≥20 000 mf/mL has been used as part of a pilot study on the efficacy of a “test-and-not-treat” (TNT) strategy in order to prevent not only the SAEs but also to reduce the incidence of marked effects (with functional impairment for several days) that could have had an impact of the adherence of the population to the strategy [20]. We focused our analyses on *L. loa* mf prevalence rates of 20 000 mf/mL and above.

**Table 1.** Transmission Matrix Relating the *Loa loa* Microfilariae (mf) Count Frequency Distribution After a Single Treatment With Ivermectin to the Pretreatment mf Count Based on Combined 6 and 12 Months Follow-up Data from Gardon et al 1997 [17]

		Fraction of Population by *L. loa* mf Intensity Category, Posttreatment						
*L. loa* mf Intensity Category, Pretreatment	Total No.	0	1–100	>100–500	>500–2000	>2000–10 000	>10 000–30 000	>30 000
0	90	0.978	0.022	0	0	0	0	0
1–100	84	0.857	0.131	0.012	0	0	0	0
>100–500	72	0.500	0.306	0.139	0.056	0	0	0
>500–2000	52	0.365	0.192	0.365	0.077	0	0	0
>2000–10 000	83	0.120	0.084	0.169	0.434	0.193	0	0
>10 000–30 000	84	0.060	0.024	0.071	0.167	0.595	0.083	0
>30 000	68	0	0	0.029	0.059	0.485	0.382	0.044

For clarity, numbers are rounded up to 3 decimal places.

Abbreviation: *L. loa*, *Loa loa*; mf, microfilariae.

### Impact of MDA on Onchocerciasis

We used the mathematical model ONCHOSIM [21–23] to predict how *O. volvulus* mf prevalence would change over time due to ivermectin mass treatment for a predefined set of different precontrol endemicity levels, up to 30 years of MDA, and various treatment coverage levels (Supplement S1, section 4.2). These simulations were used for each raster cell to define the likely trend in prevalence over time, considering the local treatment history (start date of MDA, achieved coverage, treatment frequency) as explained by Tekle et al [14]. We generated maps of the predicted prevalence of *O. volvulus* infection for 1995, 2015, and 2025.

#### Estimating the Prevalence of Coinfections

Combining estimates of the proportion of people in different *L. loa* mf intensity frequency classes and the *O. volvulus* infection prevalence, we estimated the prevalence of coinfections and, in particular, those with high *L. loa* mf intensity. By doing this per 5 km^2^ × 5 km^2^ raster cell, we accounted for any spatial correlation between the prevalence of *O. volvulus* and *L. loa*. Within each raster cell, we assumed that the probability of an individual being mf-positive for *O. volvulus* was independent of the probability that the individual was mf-positive for *L. loa*. The probability distribution per *L. loa* mf intensity class for pixels in *O. volvulus* endemic and nonendemic areas was multiplied by the total population size for 1995, 2015, and 2025. See Supplement S1, section 3.6, for more information on the source of population data.

### Uncertainty Analysis

We calculated 90% Bayesian credible intervals around the total estimated number of cases with onchocerciasis, loiasis, or coinfection with ≥20 000 *L. loa* mf/mL in the geographical area of interest (aggregated over 600 200 pixels), accounting for uncertainty in key inputs for our analysis through a Monte Carlo approach. For a detailed description of this analysis, please see Supplement S1, section 4.3, and Figure S5.

### Sensitivity Analysis

We performed a univariate sensitivity analysis to assess how results are influenced by 2 key assumptions: the impact of annual MDA on *L. loa* and the chosen critical threshold used for identifying cases with loiasis hypermicrofilaremia. Alternative assumptions for the impact of MDA on loiasis, in addition to the assumption described above, include ivermectin reduces the prevalence and intensity of *L. loa* mf after each repeated treatment with ivermectin (according to the Gardon matrix), resulting in an exponential effect of repeated treatments on *L. loa* mf, and ivermectin has no effect at all on the intensity and prevalence of *L. loa* microfilaremia. The critical threshold for identifying hypermicrofilaremic *L. loa* infections was ≥20 000 mf/mL in our baseline analysis. Alternative values considered were ≥8000 mf/mL and ≥30 000 mf/mL.

## RESULTS

### Total Number of *L. loa* Microfilaremic and Hypermicrofilaremic Cases

The total number of people living in *L. loa*-mapped areas was 81 million in 1995 and is predicted to increase to 169 million in 2025 (Table 2; Supplement S2, Table S1). For 1995, we predict that approximately 3.7 million people (4.5%) were infected with *L. loa* mf (any intensity) of whom 558 000 were infected with *L. loa* hypermicrofilaremia (0.7% of the population; 15.1% of all *L. loa* mf cases). The total absolute number of *L. loa* microfilaremic and hypermicrofilaremic cases is expected to increase to more than 6 million and 684 000, respectively, by 2025, with a respective prevalence of 3.8% and 0.4%. By 2025, the proportion of *L. loa* hypermicrofilaremics among all *L. loa*-infected cases would be 10.7%.

**Table 2.** Overview of Projections of the Number of *Onchocerca volvulus*, *Loa loa*, and Coinfected Cases for 1995, 2015, and 2025

	1995	2015	2025
Total number of cases in *Loa loa*-mapped areas			
Total population	81 331	134 823	169 257
No. (%) of people with any *L. loa* mf intensity	3698 (4.5) [3690–3714]	5017 (3.7) [4994–5039]	6382 (3.8) [6351–6412]
No. (%) of people with *L. loa* hypermicrofilaremia	558.3 (0.7) [554.2–562.4]	566.3 (0.4) [560.9–571.7]	683.8 (0.4) [676.9–691.1]
Total number of cases in *L. loa*-mapped areas that are endemic for onchocerciasis			
Areas where MDA is applied			
Total population	50 011	82 472	103 541
No. (%) of people with *Onchocerca volvulus* mf	17 156 (34.3) [17 105–17 208]	10 940 (13.3) [10 893–10 986]	982.0 (0.9) [973.4–991.2]
No. (%) of people with any *L. loa* mf intensity	2093 (4.2) [2080–2105]	2356 (2.9) [2343–2370]	2799 (2.7) [2782–2818]
No. (%) of people with *L. loa* hypermicrofilaremia	287.5 (0.6) [284.6–290.3]	118.5 (0.1) [116.3–120.8]	83.9 (0.08) [83.0–84.9]
No. (%) of coinfected cases with any *L. loa* mf intensity	865.7 (1.7) [859.1–872.1]	484.6 (0.6) [479.7–489.9]	64.1 (0.06) [62.6–65.7]
No. (%) of coinfected cases with *L. loa* hypermicrofilaremia	122.3 (0.2) [120.8–123.8]	34.2 (0.04) [33.2–35.2]	2.2 (0.002) [2.2–2.3]
Areas where MDA is not applied (hypoendemic for onchocerciasis; MDA contraindicated according to MEC/TCC guidelines because of suspected loiasis coendemicity)			
Total population	8473	13 945	17 404
No. (%) of people with *O. volvulus* mf	1612 (19.0) [1593–1629]	2651 (19.0) [2623–2682]	3302 (19.0) [3264–3338]
No. (%) of people with any *L. loa* mf intensity	508.2 (6.0) [503.1–513.3]	815.7 (5.8) [807.3–824.3]	1004 (5.8) [993.3–1014]
No. (%) of people with *L. loa* hypermicrofilaremia	81.1 (1.0) [79.6–82.7]	128.9 (0.9) [126.3–131.6]	157.8 (0.9) [154.5–160.9]
No. (%) of coinfected cases with any *L. loa* mf intensity	93.2 (1.1) [91.0–95.5]	149.7 (1.1) [146.4–153.1]	183.9 (1.1) [180.1–188.0]
No. (%) of coinfected cases with *L. loa* hypermicrofilaremia	14.9 (0.2) [14.3–15.5]	23.7 (0.2) [22.8–24.6]	28.9 (0.2) [27.9–30.0]

The percentages between parentheses in each row are based on the total number of people living in the respective areas. Absolute number of cases are provided in thousands. In square brackets are 90% Bayesian credible intervals.

Abbreviations: MDA, mass drug administration; MEC/TCC, Mectizan Expert Committee/Technical Consultative Committee; mf, microfilariae.

### Total Number of Cases in *L. loa*-Mapped Areas That Are Endemic for Onchocerciasis

A large part of the *L. loa*-mapped areas is onchocerciasis endemic; about 70% of the mapped population are expected to be in onchocerciasis-endemic areas (approximately 58.5 million people in 1995, growing to approximately 121 million in 2025), and a large part of that population (85.5%) will have likely benefited from MDA by 2025 (Table 2; Supplement S2, Tables S2–S4). We predict that the overall *O. volvulus* mf prevalence in treated areas coendemic for loiasis will substantially decrease from 34.3% in 1995 to 0.9% in 2025. We further predict a reduction in the proportion of *L. loa* mf cases over time in treated areas (from 4.2% in 1995 to 2.7% in 2025), but an increase in the absolute number (from 2.1 million in 1995 to 2.8 million in 2025) due to population growth. For *L. loa* hypermicrofilaremia, we expect a decline in both the prevalence (from 0.6% in 1995 to 0.08% in 2025) and absolute number of cases (287 000 in 1995 to 84 000 in 2025; Table 2). By 2025, we predict that about 2000 coinfected cases with *L. loa* hypermicrofilaremia will remain in treated areas. This represents only 0.002% of the total population and 0.2% of all onchocerciasis patients in these areas. A further breakdown of the number of cases by country, precontrol endemicity, and year of treatment initiation can be found in Supplement S2.

Between 1995 and 2025, the total population living in onchocerciasis-hypoendemic areas coendemic for loiasis where MDA with ivermectin is contraindicated according to Mectizan Expert Committee/Technical Consultative Committee guidelines is expected to grow from more than 8 million to 17 million (Table 2). Contrary to the situation in treated areas, we predict that the number of cases (onchocerciasis, loiasis, coinfections) will increase over time proportionally to population growth. By 2025, we expect 29 000 coinfected cases with *L. loa* hypermicrofilaremia in these hypoendemic areas if they remain untreated. This implies that 0.2% of the total population in hypoendemic areas and 0.9% of all onchocerciasis patients in these areas will be at risk of SAEs. As a result of the massive decline in the number of cases in treated areas and the increase in cases in untreated areas, we estimate that by 2025, 77.1% and 92.8% of all *O. volvulus* mf-positive cases and coinfected cases with *L. loa* hypermicrofilaremia, respectively, will live in areas where ivermectin treatment is currently contraindicated. The risk of coinfection with *L. loa* hypermicrofilaremia is predicted to remain stable in untreated areas over time.

### Onchocerciasis–Loiasis Maps

**Figure 2.**
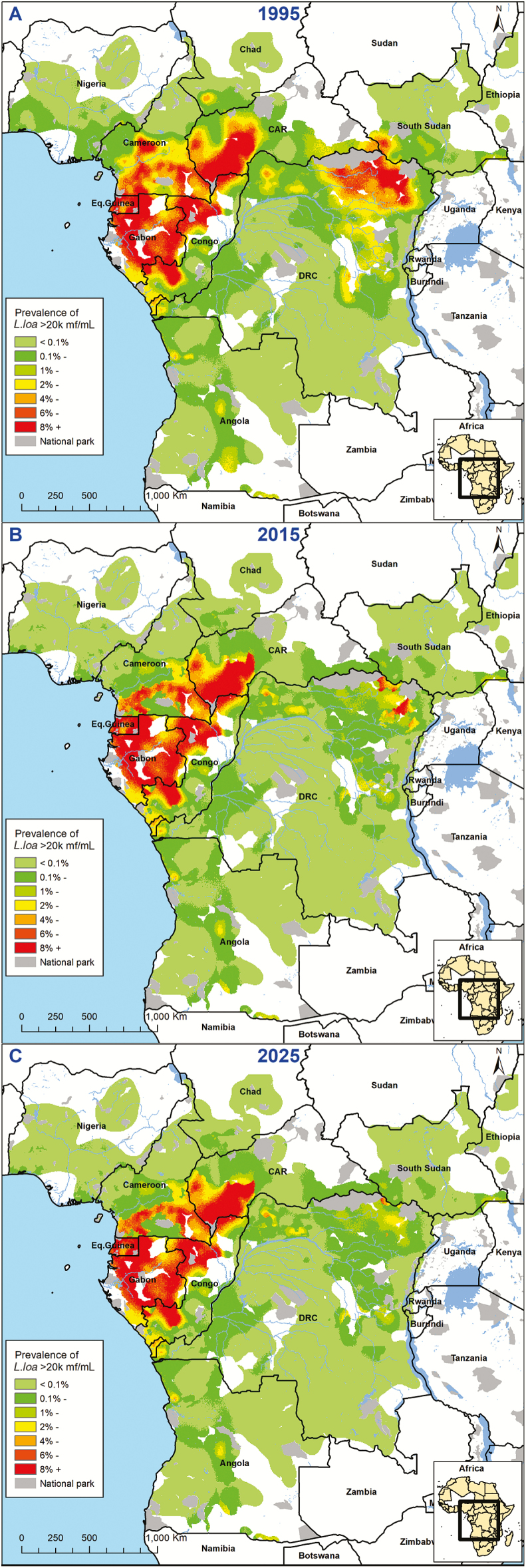
Maps showing the estimated prevalence of *L. loa* hypermicrofilaremia (≥20 000 mf/mL) in *L. loa*-mapped areas for 3 time points: 1995, precontrol (A); 2015 (B); 2025 (C). Abbreviations: CAR, Central African Republic; DRC, Democratic Republic of Congo; *L. loa*, *Loa loa*; mf, microfilariae.

Figure 2 maps the expected prevalence of hypermicrofilaremic *L. loa* cases, showing a substantial decline over time, particularly in the Democratic Republic of Congo (DRC) and Cameroon. However, we still expect sites with a hypermicrofilaremic *L. loa* prevalence of between 3% and 9% in 2025, for example, in Gabon, the Central African Republic (CAR), Cameroon, and the Republic of Congo.

**Figure 3.**
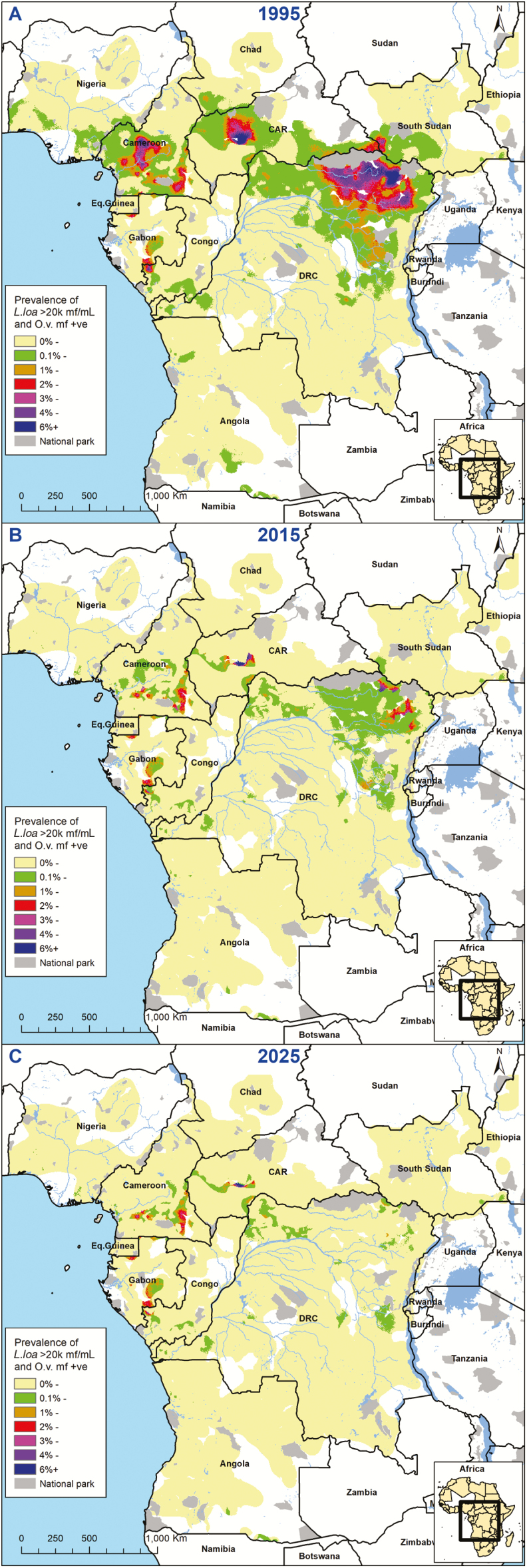
Maps showing the estimated prevalence of loiasis–onchocerciasis coinfections with *L. loa* hypermicrofilaremia (≥20 000 mf/mL) in *L. loa*-mapped areas coendemic for onchocerciasis for 3 time points: 1995, precontrol (A); 2015 (B); 2025 (C). Abbreviations: CAR, Central African Republic; DRC, Democratic Republic of Congo; IVM, ivermectin; *L. loa*, *Loa loa*; mf, microfilariae; O.v., Onchocerca volvulus.

Figure 3 shows how the estimated prevalence of loiasis–onchocerciasis coinfection with *L. loa* hypermicrofilaremia in *L. loa*-mapped areas declines over time. Precontrol, we identified hot spots of coinfected *L. loa* hypermicrofilaremic cases with prevalence rates between 6% and 12%, mainly in the Orientale province of DRC, Cameroon, and the CAR. For 2025, we predict that only some high prevalence foci will remain in the CAR, Cameroon, Gabon, and the Republic of Congo; almost all in untreated onchocerciasis-hypoendemic areas. For additional information, see Supplement S2.

### Sensitivity Analysis

**Figure 4.**
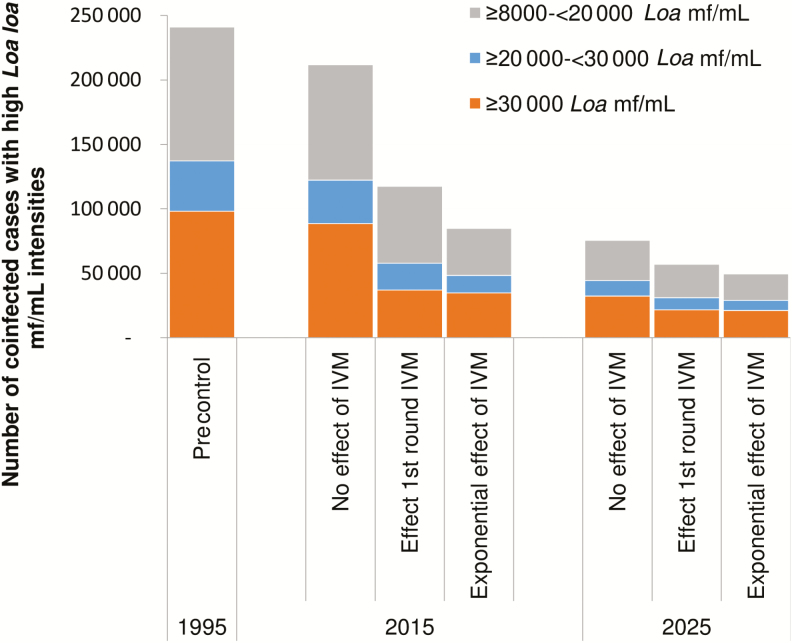
Sensitivity of the estimated number of onchocerciasis–loiasis coinfected cases by year for the following assumptions regarding the impact of mass drug administration on loiasis: *no effect of IVM:* IVM has no effect at all on the intensity and prevalence of *Loa loa* hypermicrofilaremia; *effect of first round of IVM:* IVM causes a change in the *L. loa* mf count frequency distribution among the population that received a first IVM treatment; the resulting *L. loa* mf count frequency distribution is sustained after subsequent treatment without any further changes; and *exponential effect of IVM:* IVM reduces the prevalence and intensity of *L. loa* mf after each repeated treatment with IVM, resulting in an exponential effect of repeated treatments on *L. loa* mf. Abbreviations: IVM, ivermectin; mf, microfilariae.

Figure 4 summarizes the results of the sensitivity analysis, showing the sensitivity of the predicted number of coinfected cases with hypermicrofilaremia over time with varying assumptions of the effect of MDA on loiasis. If we assume no impact of ivermectin on loiasis, the decline in the number of coinfected cases with hypermicrofilaremia is much slower than in our main analysis, and the opposite is true if we assume an exponential effect of repeated treatment. The large difference in the number of cases between the 3 assumptions in 2015 is mainly due to the fact that the highest impact of ivermectin on infection is achieved after a first MDA round with ivermectin (both on *L. loa* intensity and prevalence), which obviously did not occur in the scenario of no effect of ivermectin on *L. loa*. However, the number of cases under the latter assumption was predicted to decline more dramatically between 2015 and 2025, mainly thanks to the decline in onchocerciasis in the coinfected cases. Consequently, little difference remains between the assumptions in the predicted number of *L. loa* hypermicrofilaremic coinfected cases by 2025.

## DISCUSSION

We anticipate major reductions in the prevalence of *O. volvulus* (89.1%) and coinfection cases with *L. loa* hypermicrofilaremia (85.0%) between 1995 (precontrol) and 2025 in areas that are coendemic for loiasis and currently subject to ivermectin MDA (Table 2; Supplement S2, Table S2). We predict that by 2025, untreated onchocerciasis-hypoendemic areas will contain 77.1% of all remaining onchocerciasis-infected individuals, 65.3% of all *L. loa* hypermicrofilaremic cases, and 92.9% of all coinfected *L. loa* hypermicrofilaremic cases. Adapted policy recommendations are required for these areas.

An important policy question is what needs to be done in currently untreated areas. Some areas in our study are thought to be free of onchocerciasis (eg, untreated areas in Burundi, Chad, and Equatorial Guinea [24] but also scattered patches in some other countries). If this is confirmed by further elimination mapping, no onchocerciasis control measures are required. We also identified several onchocerciasis-hypoendemic areas in Ethiopia that are likely to be free from loiasis. If this is confirmed by further mapping, one can proceed with standard MDA. However, most problematic are onchocerciasis-hypoendemic areas coendemic with loiasis where a proportion of the population is still *L. loa* hypermicrofilaremic. For these areas, a safe option would be to implement a TNT strategy aimed at excluding severely infected individuals from treatment through testing of peripheral blood with a videomicroscope (LoaScope) [20]. According to our calculations, in 2015 about 14 million people lived in areas where TNT might be a suitable strategy for moving forward with onchocerciasis elimination, of which 129 000 (0.9%) would be at high risk of SAEs following ivermectin treatment. The prevalence of *L. loa* hypermicrofilaremia varies between onchocerciasis-hypoendemic areas, with predicted prevalences of <0.2% in Sudan and Nigeria and >5.0% in Gabon and the CAR. These results are in line with other published estimates [25–27]. In a recent TNT trial in Cameroon (2015 data), 2.1% of the screened individuals were excluded from ivermectin distribution due to *L. loa* mf density ≥20 000 mf/mL [20]. In our analysis, we predict that for *L. loa*-endemic onchocerciasis-hypoendemic areas in Cameroon, 3.9% of the population would need to be excluded from ivermectin during a TNT on the basis of *L. loa* mf density ≥20 000 mf/mL blood. A TNT strategy is costlier than standard MDA [28], which raises questions about the affordability of implementing TNT on a wide scale. A strategy for eliminating onchocerciasis in hypoendemic areas could potentially include community-directed vector control (eg, slash and clear vegetation [29], ground-based larviciding) to supplement TNT-based medication.

New or adapted safe drugs with macrofilaricidal activity that can potentially be used for mass distribution in *L. loa* coendemic areas should be considered. Current second-line treatments for usage in hypermicrofilaremic coinfected individuals have limitations, that is, doxycycline needs to be taken daily for 4 to 6 weeks, is age-restricted, and cannot be used in pregnant and breast-feeding women [30, 31], whereas moxidectin [32] is also likely contraindicated in patients with heavy *L. loa* infections due to the risk of SAEs.

We estimate that by 2025 there will be more than 6 million *L. loa* microfilaremic cases remaining that may require treatment for loiasis infection. *Loa loa* may cause more harm than commonly suggested; infection can lead to various severe complications, including cardiac fibrosis, encephalopathy (in the absence of treatment), pulmonary infiltrates, neurological and psychiatric disorders, and excess mortality [33, 34]. We predict that by 2025, 684 000 people living in nonendemic areas for onchocerciasis will be *L. loa* hypermicrofilaremic, justifying additional investments in research and drug development for treating *L. loa* infection.

The results presented here are estimates based on the most comprehensive available data, yet there are some uncertainties and assumptions. Our analysis uses baseline data (REMO and RAPLOA) that are relatively old. The distribution of infection and population may have changed in the meantime. Each of the 2 rapid assessment surveys have their own specific challenges (eg, mode of assessment, selection of survey sites and participants, sample size requirements) [4, 35] that may have led to some imprecision in our estimates for certain geographical areas, for example, some previously identified hypoendemic areas might be free of onchocerciasis [36], but we expect that the main conclusion will remain the same.

ONCHOSIM simulated the impact of ivermectin in treated areas according to reported treatment history and future MDA scenarios. Although current mathematical models capture community infection dynamics throughout MDA effectively, any incorrect assumptions on MDA initiation year, overreporting of MDA coverage, or MDA implementation challenges would result in higher *O. volvulus* mf and coinfected case estimates for 2015 and 2025. Similarly, based on a published transition matrix, we assume that MDA impacts *L. loa* mf intensity and prevalence [17]. The robustness of these assumptions was assessed through a sensitivity analysis and found to be in line with evidence from a recent study on the impact of repeated annual MDA with ivermectin on loiasis prevalence and intensity in Cameroon [19]. These data from Cameroon suggest that repetitive use of ivermectin may exponentially impact *L. loa* intensity (and to a lesser extent prevalence), with the highest effect after the first round of treatment. Finally, in this analysis, we have not taken into account any spatiotemporal covariates, such as the impact of climate change or deforestation on *Simulium* spp. and *Chrysops* spp. vectors. Additional data on these covariates and their impact on vector density and infection levels could further improve our predictions.

## CONCLUSIONS

According to our estimates, MDA has a remarkable impact on onchocerciasis and loiasis coinfected cases across Africa since the start of mass distribution of ivermectin. The highest number of remaining coinfected cases with *L. loa* hypermicrofilaremia will be in onchocerciasis-hypoendemic areas that would benefit most from alternative treatment strategies, such as TNT or alternative treatments.

## Supplementary Data

Supplementary materials are available at *Clinical Infectious Diseases* online. Consisting of data provided by the authors to benefit the reader, the posted materials are not copyedited and are the sole responsibility of the authors, so questions or comments should be addressed to the corresponding author.

ciz647_suppl_Supplement_Information_S1

ciz647_suppl_Supplement_Information_S2

ciz647_suppl_Supplement_Information_S3

## Notes

***Author contributions.*** N. V. S. V. M., L. E. C., B. P., and W. A. S. were responsible for the conception of the study. N. V. S. V. M., L. E. C., M. B., B. P., S. D. S. P., J. H. R., and W. A. S. were responsible for the design of the analysis. J. H. R. performed the analysis. N. V. S. V. M. wrote the first draft of the manuscript. All coauthors contributed substantially to the interpretation of the results, critical revision of the manuscript for important intellectual content, and approved the final version of the manuscript for publication. All authors were aware of and agreed to be accountable for all aspects of the work in ensuring that questions related to the accuracy or integrity of any part of the work are appropriately investigated and resolved.

***Acknowledgments.*** The authors warmly thank Louise Burrows (Drugs for Neglected Diseases *initiative* [DND*i*]) for editing the final draft of the manuscript. The authors acknowledge the World Health Organization Regional Office of Africa (WHO–AFRO) for approving the use and publication of the data used in this article.

***Disclaimer.*** B. P. works at the organization that sponsored this study. The contents do not necessarily reflect the views of United States Agency for International Development (USAID), the US government, or WHO. The contents are the responsibility of the Department of Public Health, Erasmus MC, University Medical Center Rotterdam (the Netherlands). All authors agree that the study sponsor did not have any influence on the study design, analysis, interpretation of results, the writing of the manuscript, and the decision to submit the manuscript for publication, other than the critical, independent, and scientific contribution of a senior researcher. The pixel-level, raw data are in possession of WHO–AFRO, but the corresponding author has full access to the aggregated project-level data on the number of cases for the 3 time points. The corresponding author had final responsibility for the decision to submit for publication.

***Financial support.*** N. V. S. V. M., W. A. S., L. E. C., and B. P. received funding from USAID through the DND*i*) (grant AID-OAA-G14-00010). W. A. S., S. D. S. P., M. B., and S. W. acknowledge financial support from the TNT grant (Bill & Melinda Gates Foundation, ref. no. Nr OPP1033740). W. A. S. and L. E. C. also acknowledge financial support from the NTD Modelling Consortium (Bill & Melinda Gates Foundation, OPP1184344). In addition, L.E.C. was financially supported through an Innovational Research Incentives Scheme Veni by the Dutch Research Council (NWO, grant 016.Veni.178.023). A. H. T. and H. F. M. Z. were financed by WHO.

***Potential conflicts of interest.*** All authors report no potential conflicts of interest. All authors have submitted the ICMJE Form for Disclosure of Potential Conflicts of Interest. Conflicts that the editors consider relevant to the content of the manuscript have been disclosed.
