# Supplementary material for: Projected Number of People With Onchocerciasis–Loiasis Coinfection in Africa, 1995 to 2025
Source: Clin Infect Dis. 2019 Jul 13;70(11):2281–9. doi: 10.1093/cid/ciz647 (PMC7245158; doi:10.1093/cid/ciz647)
Supplement: ciz647_suppl_Supplement_Information_S1 [file ciz647_suppl_supplement_information_s1.pdf]

Projected number of people with onchocerciasis-loiasis co-infection in Africa, 1995 to 2025

Supplementary information S1: Detailed methods

Natalie VS Vinkeles Melchers[1#], Luc E Coffeng[1], Michel Boussinesq[2], Belén Pedrique[3], Sébastien DS Pion[2], Afework H Tekle[4], Honorat GM Zouré[5], Samuel Wanji[6], Jan Remme[7*], Wilma A Stolk[1*]
* Contributed equally

[1] Department of Public Health, Erasmus MC, University Medical Center Rotterdam, P.O. box 2040, 3000 CA Rotterdam, The Netherlands
[2] Unité Mixte Internationale 233 « TransVIHMI », Institut de Recherche pour le Développement (IRD), INSERM U1175, University of Montpellier, Montpellier, France
[3] Drugs for Neglected Diseases *initiative*, 15 Chemin Louis Dunant, 1202 Geneva, Switzerland
[4] Preventive Chemotherapy and Transmission Control Unit, Control of Neglected Tropical Diseases Department, World Health Organization, Geneva, Switzerland
[5] ESPEN, World Health Organization, Regional Office for Africa, Cité du Djoué, Brazzaville, Republic of Congo
[6] Parasites and Vectors Research Unit, Department of Microbiology and Parasitology, University of Buea, Cameroon
[7] 120 Rue des Campanules, Ornex, France

*Correspondance to:
Natalie VS Vinkeles Melchers, Department of Public Health, Erasmus MC, University Medical Center Rotterdam, P.O. box 2040, 3000 CA Rotterdam, the Netherlands
n.vinkelesmelchers@erasmusmc.nl, Natalie.melchers@gmail.com; +31 (0)10 70 38465

Table of Contents

1 Definitions ... 3

2 Study outcomes ... 4

3 Data and analysis ... 5

3.1 Geographic scope of the analysis ... 5

3.2 Pre-control loiasis endemicity map ... 6

3.3 Pre-control onchocerciasis endemicity map ... 10

3.4 Conversion from prevalence of history of eye worm to L. loa mf intensity ... 11

3.5 APOC treatment database ... 14

3.6 Population size per geographical area ... 15

4 Predicted impact of ivermectin MDA ... 15

4.1 Impact of MDA on the frequency distribution of L. loa mf counts ... 15

4.2 Impact of MDA on onchocerciasis ... 17

4.3 Uncertainty analysis ... 18

5 Mathematical and statistical programmes ... 23

6 References ... 24

1 Definitions

Table S1. List of definitions used in the manuscript**.**

Terminology	Definition
**Infected person**	Someone who is positive for either *O. volvulus* or *L. loa* microfilariae.
**Co-infected person**	Someone who is positive for both *O. volvulus* and *L. loa* microfilariae.
***L. loa* hypermicrofilaraemia**	Individuals with *L. loa* mf loads of ≥20,000 mf/mL blood, who are considered to be at high risk of SAEs following ivermectin treatment.
**Classification of *L. loa* mf/mL intensity**	We defined a classification of *L. loa* mf loads (≥8,000 - <20,000 mf/mL; ≥20,000 - <30,000 mf/mL; ≥30,000 mf/mL).
***L. loa*-mapped population**	The extrapolated population living in the area mapped by RAPLOA for loiasis (6.9 million km^2 in 14 countries).
**Population in *L. loa*-mapped areas that are endemic for onchocerciasis**	The predicted population living in areas that were mapped for loiasis (see above) and are endemic for onchocerciasis.
**APOC project**	A geographic area taken as the implementation unit for community-directed treatment with ivermectin (CDTi). Each APOC project has its own organisational structure responsible for implementing the recommended CTDi strategy [1]; a list of all APOC projects is available elsewhere [2].
**P5 project**	A combination of all areas in a country that are considered to be hypoendemic for onchocerciasis (based on REMO surveys showing nodule prevalence levels between 5% and 20%), that were not part of previously defined APOC projects, and that are likely to require treatment or other interventions for the purpose of onchocerciasis elimination.
**P20 project**	A combination of all areas in a country that are considered to

	be hyper- and mesoendemic for onchocerciasis (based on REMO surveys showing nodule prevalence levels of ≥20%), that were not part of previously defined APOC projects, and that are targeted for MDA for the purpose of onchocerciasis elimination.
**Pre-control onchocerciasis endemicity classes**	Categories used to group APOC projects based on the highest predicted pre-control nodule prevalence among adult males in the project area: non-endemic (<5%), hypoendemic (≥5% - 20%), mesoendemic (≥20% - <40%), and hyperendemic (≥40%) [3–5].

2 Study outcomes

The primary study outcome is the total number of loiasis-onchocerciasis co-infected cases with very high *L. loa* mf counts (≥20,000 mf/mL) for 1995, 2015 and 2025. The secondary study outcomes are i) the total number of *L. loa* microfilaraemic and hypermicrofilaraemic cases (independent of presence of onchocerciasis); ii) the total number of onchocerciasis cases (independent of presence of loiasis); and iii) the total number of people to be screened in the case of a Test-and-Not-Treat programme in onchocerciasis hypoendemic areas potentially co-endemic for loiasis.

3 Data and analysis

3.1 Geographic scope of the analysis

The geographical scope of this analysis includes areas that were surveyed for *L. loa* endemicity across countries previously under the African Programme for Onchocercisis Control-mandate (APOC-countries) using the RAPLOA procedure. These areas were selected on the basis of environmental risk models using remote sensing data for Central and West Africa, which predicted the prevalence of *L. loa* infection using environmental covariates that are associated with a suitable environment for the presence of *Chrysops spp* [6,7]. Countries and APOC-projects were not surveyed in their entirety (see Figure S1).

Figure S1. Map of area for *L. loa* and onchocerciasis overlap used in the analysis.

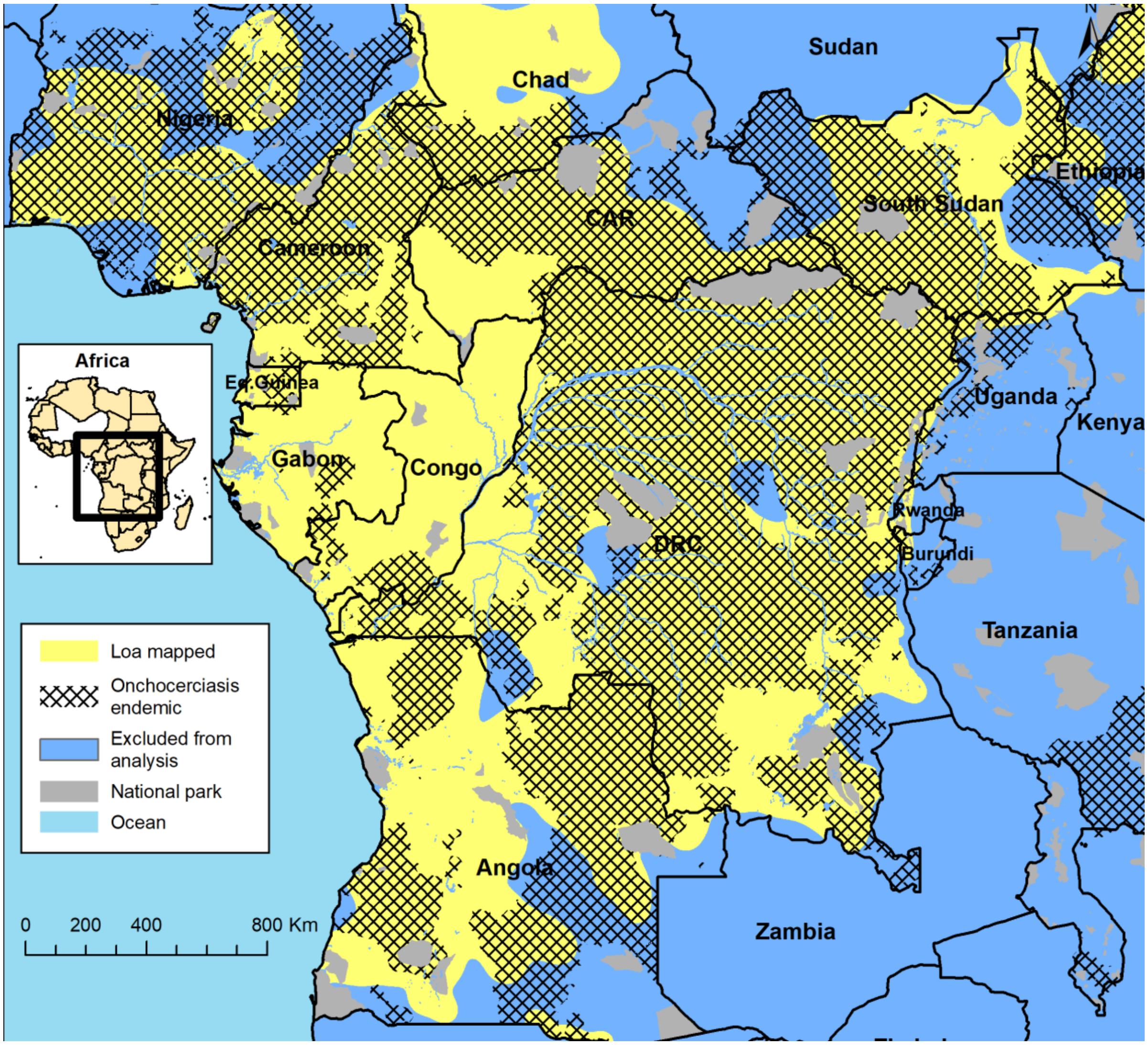

3.2 Pre-control loiasis endemicity map

We used loiasis prevalence maps based on "Rapid Assessment Procedure for Loiasis" (RAPLOA) surveys that were performed by APOC to identify areas potentially endemic for *L. loa*, excluding uninhabited areas (e.g. national parks, swamps) [8–10]. RAPLOA is a simple method assessing loiasis prevalence from a sample of the population using a short questionnaire asking about history of eye worm (i.e. migration of the adult *L. loa* under the eye conjunctiva). RAPLOA data were collected in 5,158 villages covering all areas with suspected loiasis endemicity, irrespective of onchocerciasis endemicity. A loiasis prevalence raster map was previously generated by geostatistical kriging analysis of the RAPLOA data for all potentially endemic areas in 14 countries [11].

We updated this map to capture new unpublished data from Nigeria and Angola, repeating the original geostatistical analysis (using the same kriging methodology) on the combined data set of 5,158 surveyed villages. The geostatistical analysis covers some border areas of countries where no RAPLOA surveys were done, but kriging could be performed (Burundi, Sudan, Uganda). Weighted spatial smoothing was used to predict the distribution of *L. loa* mf prevalence, with highest precision close to RAPLOA-sampled locations. Smoothing of the map was still precise up to a maximum of a 100 km radius around the nearest sampled village, the maximum distance depending on the number of villages sampled, as assessed by others [11]. A "RAPLOA-surveyed area" in this study was therefore defined as all areas within a 100 km radius around sampled villages. Small border areas in Burundi, Sudan and Uganda were within this maximum radius of 100 km to a nearest sampled village in an onchocerciasis-endemic bordering community, and henceforth prevalence predictions were performed for these countries with good precision. The original satellite mapping of *L. loa* prevalence by Thomson *et al.* [7], on which the selection of RAPLOA-surveyed areas was based, did not include risk mapping of Burundi, Sudan and Uganda. According to our results, there are very few *L. loa* hypermicrofilaraemic cases in these border areas (<200 cases) with negligible prevalence rates; only the onchocerciasis hypoendemic border area in Sudan has a predicted prevalence of 0.08% *L. loa* hypermicrofilaraemia, Burundi and Uganda were found to be negative.

The original RAPLOA map was based on RAPLOA surveys performed between 2002-2006 (phase 1) and 2008-2010 (phase 2) in nearly all areas in APOC-countries that were assumed to be potentially endemic for *L. loa* [11]. Areas that remained to be mapped were Nigeria and Angola, as well as some border areas between north-eastern CAR and north-western South Sudan (low population density but likely to be highly endemic for loiasis) that could not be mapped due to insecurity concerns [11] (Fig. S1, onchocerciasis-endemic areas of the CAR and western South Sudan that are not *L. loa*-mapped). The pre-control onchocerciasis REMO map shows moderate to high nodule prevalence rates, and it is important to first perform onchocerciasis elimination mapping, before assessing *L. loa* presence, once these areas are safe to enter.

In much of Angola, the planned surveys could not be completed before the end of 2010. These areas were not included in the original RAPLOA map but the remaining RAPLOA surveys in Angola were performed in 2011 among 114 villages. Furthermore, additional RAPLOA surveys were performed in 2014 among 238 villages in Nigeria to validate some of the original RAPLOA results and confirm the presumed absence of loiasis from other parts of the country (Table S2). There are no methodological differences between the RAPLOA surveys performed in Angola and Nigeria and phase 2 RAPLOA surveys. Between phase 1 and phase 2 RAPLOA surveys, the distance between sample villages was gradually increased from 10 km to 25 km as the distribution of loiasis was found to be more homogenous than initially thought [11]. These additional RAPLOA survey results for Angola and Nigeria have now been added to our analysis and we have updated the RAPLOA map (Figure S3) using the same kriging methodology as described by Zouré *et al.* [11].

Table S2. Number of villages surveyed and history of eye worm in Angola and Nigeria.

Country	No. villages surveyed	No. of people interviewed	Mean no. interviewed per village	No. (%) interviewed who had history of eye worm	Percentage with eye worm history		
					Min.	Median	Max.
Angola	114	8,675	76.1	985 (11.4%)	0.0	5.0	83.3
Nigeria	238	18,958	79.7	1,354 (7.1%)	0.0	3.8	55.0

The REMO and RAPLOA maps are the most detailed maps currently available on the prevalence of onchocerciasis and loiasis in the APOC-countries. Between 2000-2004, several geospatial models were used to develop environmental risk maps for *L. loa* based on land and forest cover, soil type, Normalised Difference Vegetation Index and elevation [6,7,12]. RAPLOA surveys were performed after the publication of these environmental risk maps. Several important discrepancies were found between the environmental risk maps and the RAPLOA map [11]. Although environmental risk models have proved useful tools for indicating the general spread of infection, they are not considered reliable enough for decision-making on ivermectin treatment. For onchocerciasis, there is evidence that the distance between villages and rivers (breeding sites) may be an important environmental predictor for *O. volvulus* infection in West-Africa (countries originally under the Onchocerciasis Control Programme-mandate (OCP)). Other environmental covariates were found to play little or no role in predicting the distribution of onchocerciasis [13].

Similarly, weak evidence of environmental predictors on *L. loa* transmission are found elsewhere [6,7]. Little effect of deforestation is expected on our estimates, as most *L. loa* transmission occurs by the vector *C. silacea*, and it was found that its dominance in villages sustains transmission even with forest clearance [14]. Henceforth, the spatial models for onchocerciasis and loiasis in this analysis are based on extensive survey data and do not include environmental covariates. Alignment of this analysis with published environmental

models [15–17] could improve prevalence predictions of these diseases for the assessment of areas at risk of SAEs.

Figure S2. Updated map of the estimated prevalence of eye worm history in Africa (including Angola and Nigeria), based on the Rapid Assessment Procedure for Loiasis (RAPLOA) surveys carried out between 2002 and 2014.

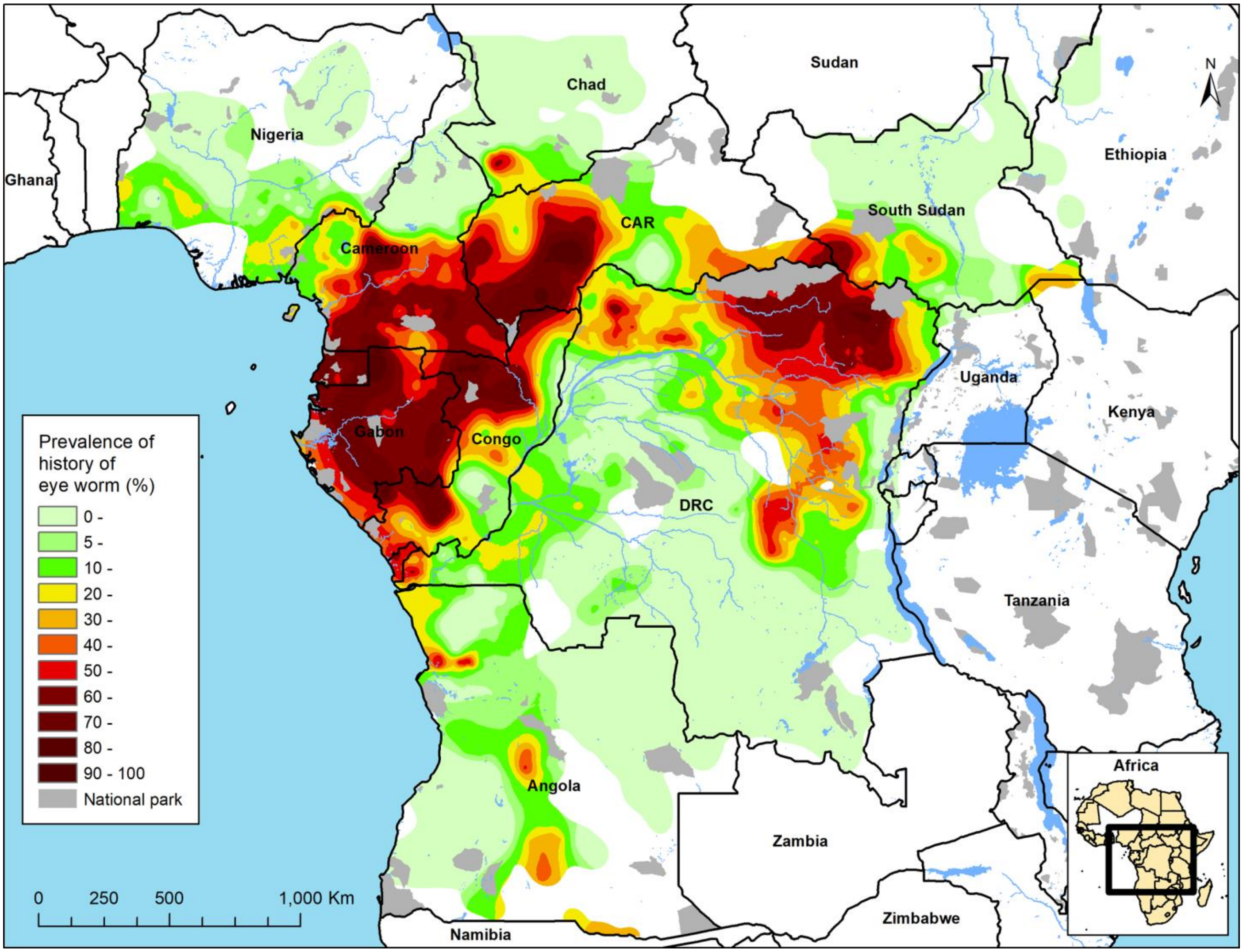

3.3 *Pre-control onchocerciasis endemicity map*

Our analysis is based on a previously published, high-resolution map (5x5 km^2 raster) of the pre-control prevalence of onchocerciasis nodules, that was generated by a geostatistical analysis of data from Rapid Epidemiological Mapping of Onchocerciasis (REMO) surveys (see Figure S2) [18]. REMO surveys were carried out by APOC in a spatial sample of >14,000 villages, examining 30-50 adults for infection with onchocerciasis by assessing the presence of sub-cutaneous onchocercal nodules.

Figure S3. Map of the estimated pre-control (1995) prevalence of palpable nodules among adult males (>20 years of age) in 20 APOC-countries, based on the Rapid Epidemiological Mapping of Onchocerciasis (REMO) [18].

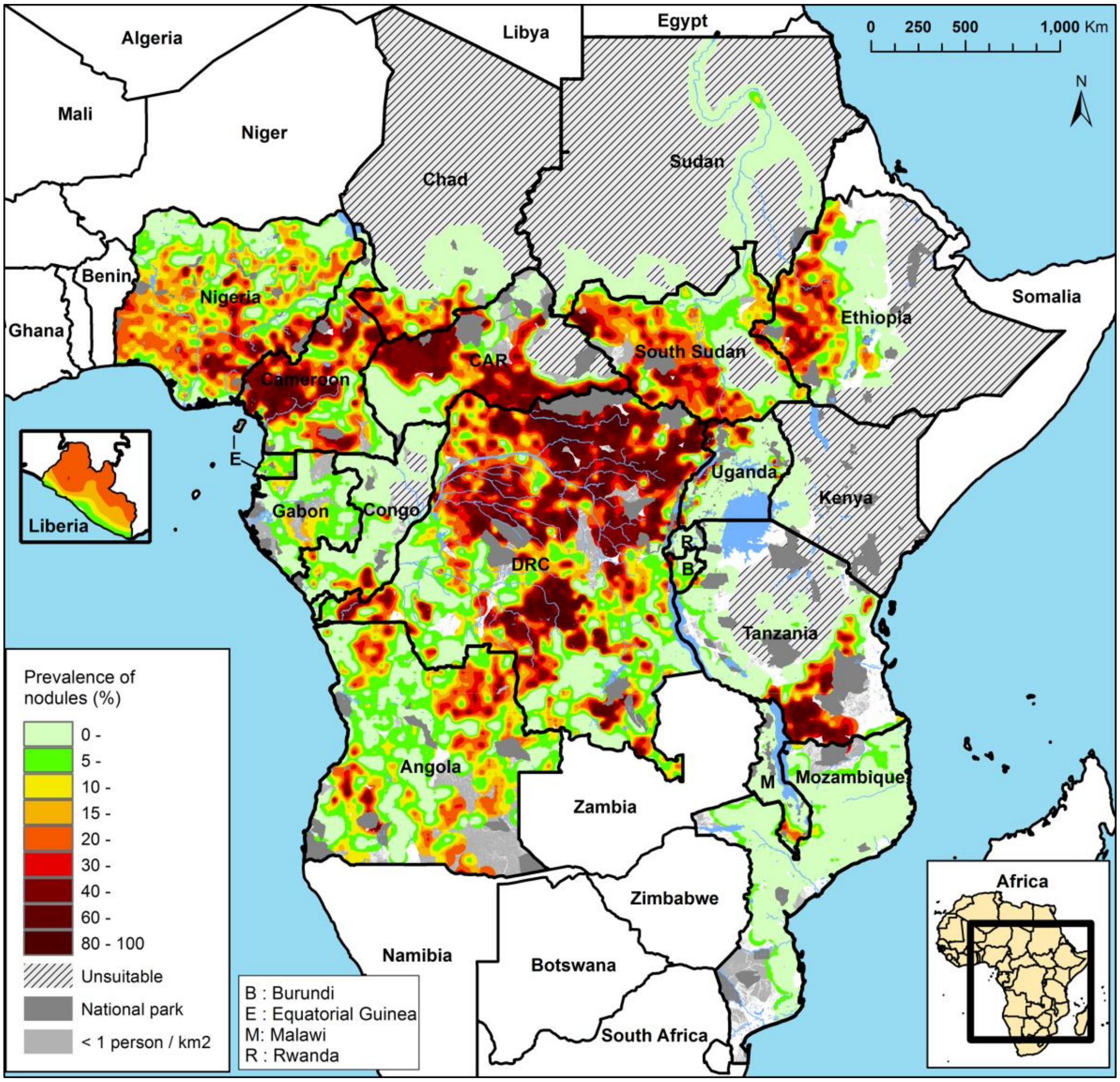

A somewhat arbitrary classification of hypoendemic areas is used based on nodule prevalence with a cut-off point of 5% (see Table S1), assuming that areas with 5% to 20% nodule prevalence are hypoendemic transmission areas which should be treated according to current guidelines. Further validation of infection/transmission in areas below 20% nodule prevalence is required before treatment decisions are made. REMO has historically been used for delineation of treatment areas in Africa, and APOC considered this classification as indicative only. The rapid assessment was mainly used for prioritisation of areas in need of MDA due to too high risks of severe onchocercal disease, which occurred when the *O. volvulus* mf prevalence was above 35% (corresponding to a nodule prevalence of 20%) [19]. Onchocerciasis elimination requires identification and treatment of geographical areas that can sustain local transmission, and empirical and modelling studies indicate that transmission is unstable or will decrease to local extinction in areas of less than 20% *O. volvulus* mf prevalence (≈10% nodule prevalence) [20].

3.4 Conversion from prevalence of history of eye worm to L. loa mf intensity

Data

To translate the prevalence of history of eye worm into the prevalence of *L. loa* hypermicrofilaraemia, we developed a statistical model based on data from the original RAPLOA studies, using the subset of data from villages for which both history of eye worm and the intensity of *L. loa* infection measured in 20 μL blood was available for each individual participating in the study. We used data from multiple countries in Africa, i.e. Cameroon, Nigeria and DRC [8–10]. We used data sources from 11 bio-geographical zones (covering forest, intermediate and savanna bioclimes) from the original study that developed RAPLOA [8,10], and the subsequent validation study in DRC that re-assessed the previously assessed functional relationship between the prevalence of high intensity loiasis infection and the prevalence of *L. loa* mf. These communities were sampled from eastern to western highly *L. loa* endemic areas of Africa, and comprised communities with a heterogeneous range of RAPLOA prevalence in Cameroon, Nigeria and DRC. The applied functional relationship is therefore representative for the majority of *L. loa*-endemic Africa.

The original RAPLOA study also covered a bio-geographical zone in Nigeria, where the prevalence of loiasis was very low (RAPLOA prevalence of <40%, corresponding to a *L. loa* mf prevalence of <2%). A recent study in southern Nigeria assessed the *L. loa* mf density in the blood using the LoaScope in communities of low, intermediate, and high RAPLOA prevalence rates.[21] It is challenging to compare the data from Emukah *et al.* [21] with those from the RAPLOA surveys, due to methodological differences, e.g. their usage of the Loascope (which is not specific for *L. loa*) as a diagnostic tool for measuring mf prevalence, different age groups (almost 50% of the sampled individuals were children), and purposive village sampling (75% of all sampled villages were <40% RAPLOA prevalence). It is therefore difficult to judge whether the association between high loiasis prevalence and low density blood mf as suggested in the study by Emukah *et al.* [21] is statistically different from the association used by us. Even so, we expect that applying an adapted functional relationship for Nigeria based on the study by Emukah *et al.* in our model would have little impact on our results, as we already predict very low prevalence rates (0.1%) of loiasis hypermicrofilaraemia among the population in hypoendemic areas of Nigeria for 2015.

Statistical model

The statistical model comprises a bivariate normal distribution describing the joint distribution of village-level logit-prevalence of history of eye worm and the log-mean *L. loa* mf intensities (Figure S4). Further, we assumed that within each village, individual *L. loa* mf counts follow a negative binomial distribution with a shape parameter *k*, where the logarithm of *k* increases linearly with the log-mean *L. loa* mf intensity in the village (i.e. such that over-dispersion of individual *L. loa* mf counts increases with lower mean *L. loa* infection levels in a village). Model parameters and parameter uncertainty were quantified in a Bayesian framework using dynamic Hamiltonian Monte Carlo. Then, given a pixel-level estimate of the prevalence of history of eye worm from the RAPLOA map, we used the statistical model to predict the conditional distribution of the mean intensity of infection and the associated value of *k* for that pixel, which in turn was used to predict the pre-control prevalence of different categories of *L. loa* mf counts.

Figure S4. Statistical association between RAPLOA prevalence (x-axis) and prevalence of different categories for intensity of *L. loa* infection (y-axis). Symbols represent village-level data from Nigeria, Cameroon, and DRC [8–10]. Solid represent model expectation (posterior mean); the darker shaded area represents the 90%-Bayesian credible interval (BCI) for the posterior mean; the lighter shaded area represents the 90%-BCI for individual village-level predictions given a (point-)estimate of the RAPLOA prevalence. The association was used to translate individual pixel-level draws of the RAPLOA prevalence to village-level predictions for prevalence of the different intensity categories.

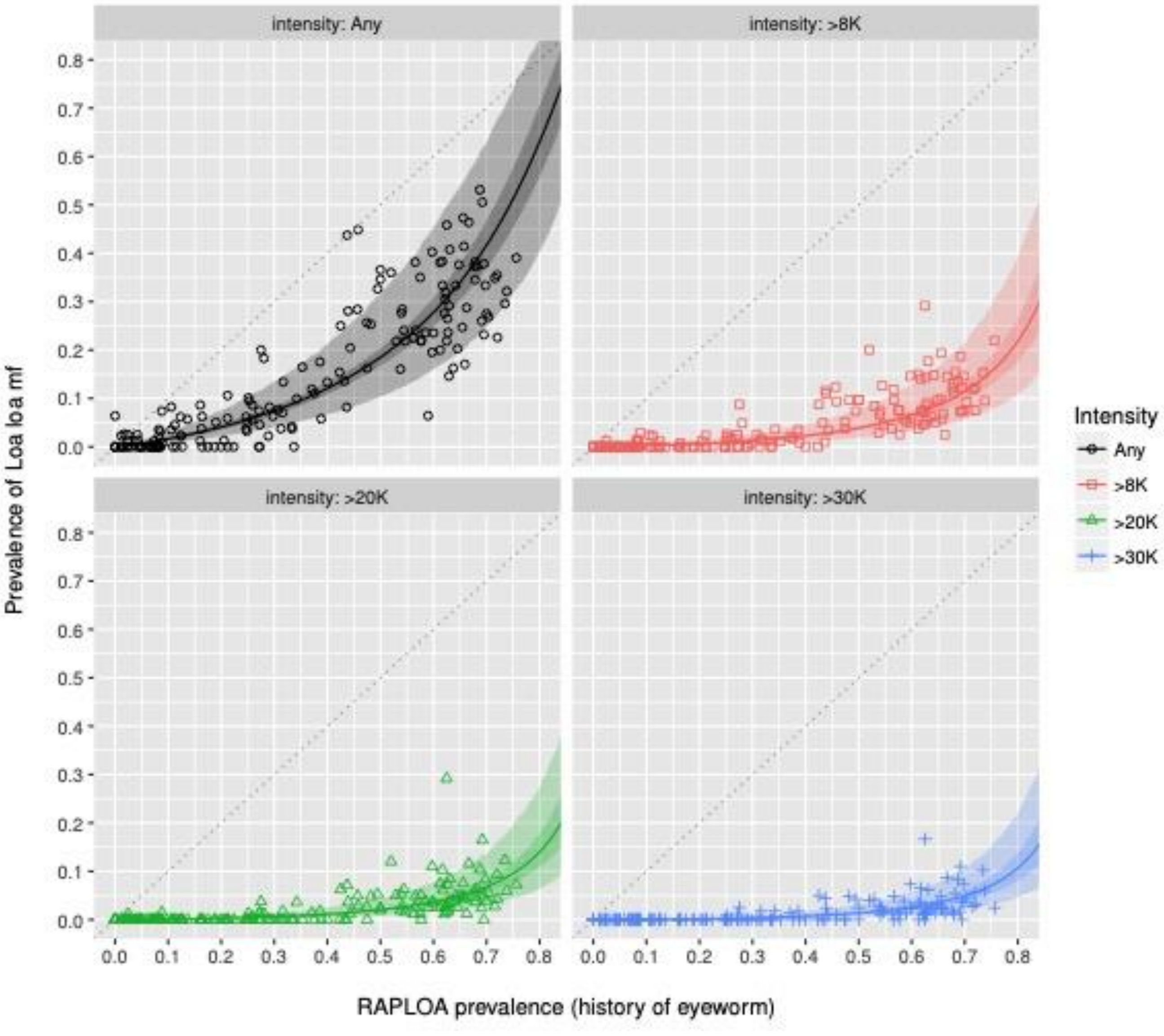

3.5 APOC treatment database

After we converted the geographical map of all APOC-projects into a 5x5 km^2 raster file, we linked it to project-specific data on treatment history (1995-2013) and project-specific scenarios of expected treatments in the period 2014-2025. Treatment history data up to November 2013 were obtained from Kim *et al*. [2], and included the number of treatment rounds per year and population coverage. We assumed that MDA would not be introduced before 2025 in the so-called "P5" hypoendemic areas that are potentially co-endemic for loiasis. MDA should not be implemented in such areas due to risk of SAEs [22].

There are several APOC-projects where MDA had not yet started by 2013, which are called "Not Yet" (NY) projects (as they are not yet treated with ivermectin), and have previously been listed by Kim *et al.* [2]. Although Kim *et al.* classified some of these NY areas as hypoendemic, they were found to be mesoendemic by our model-based geostatistics and were classified by APOC as high risk/requiring treatment [19]. In our analysis, these projects are assumed to start with MDA before 2025 in spite of the potential presence of loiasis. Supplement S3 provides more information on the reported and assumed treatment history per APOC-project. Likewise, all other meso- and hyperendemic areas that have not yet initiated MDA by 2013 (up to which year treatment history is available, as published by Kim *et al.*[2]) are assumed to have started between 2014 and 2016. This is in accordance with the modified strategies as outlined in the *Mectizan Expert Committee/Technical Consultative Committee* guidelines [23], advising MDA in all onchocerciasis hyper- and mesoendemic areas, as the benefits of treating onchocerciasis are considered to outweigh the risk of *L. loa*-related SAEs. Specific surveillance systems should be considered for recognition and case management of SAE. All treated areas were assumed to continue MDA until at least 2025, except on the island of Bioko (Equatorial Guinea), where onchocerciasis was eliminated [24].

3.6 Population size per geographical area

We obtained the pre-control (1995) population size of each APOC-project from the APOC treatment database. The population size per APOC-project was previously collected through a census conducted by community drug distributors for estimating the amount of ivermectin required in mass treatments. For projects where such information was not available, the population size was estimated by multiplying the project's surface area with the average population density (per km^2) in the other APOC-projects within the country (similar to the Kim *et al.* methodology [2]). The size of the population at risk per country for the years 2015 and 2025 were based on the initial population at risk in 1995, and multiplied by country-specific population growth rates calculated up to 2015 as reported by the UN Population Division [25]. This growth rate was then also applied to calculate the country-specific population at risk for 2025. For RAPLOA-surveyed areas outside APOC-projects, we used a raster map of rural population density in Africa from the International Food Policy Research Institute [26].

4 Predicted impact of ivermectin MDA

4.1 Impact of MDA on the frequency distribution of L. loa mf counts

There is some evidence of an impact of ivermectin on *L. loa* mf prevalence and intensity. Gardon *et al.* found that a single dose of ivermectin may result in a reduction in *L. loa* mf intensity lasting for one year, depending on pre-treatment mf loads [27], while repeated three-monthly doses seem to have a cumulative effect on *L. loa* mf prevalence and intensity [28]. The effect of annually repeated treatment on *L. loa*-mf prevalence and intensity has also been reported [29]. The change in *L. loa* mf count frequency distribution due to annual treatment was modelled using a Markov transition matrix for the year-to-year change in proportion of the population in pre-defined *L. loa* mf intensity classes (Table 1 main text). This transition matrix was derived from pre-control data published by Gardon *et al.* [27]. We combined the data for the two sites (6 and 12 months after treatment) as there was no significant difference in the pattern of response to ivermectin between the two districts. In the data from Gardon *et al.*, which were collected in settings with high loiasis endemicity, a

small proportion of individuals with low *L. loa* mf counts before treatment moved to a higher *L. loa* mf intensity class after treatment. This transition is unlikely to occur in settings with low *L. loa* endemicity. MDA coverage was taken into account in the *L. loa* prevalence and intensity estimates through an assumed random participation to MDA, such that the matrix by Gardon *et al.* could by applied to a random fraction of the population that participated to MDA programmes. For the scenario of cumulative impact of MDA, we assumed random participation such that individuals who did not participate in the first round of MDA, would have equal chance to participate in a second round of MDA. As input for estimating the various *L. loa* mf intensity classes, we used the matrix of Gardon *et al.* who looked at seven categories of *L. loa* mf intensity (0, 1 - 100, >100 - 500, >500 - 2,000, >2,000 - 10,000, >10,000 - 30,000, >30,000) (see Table 1 in main manuscript). We therefore first applied these seven categories for our predicted *L. loa* intensity distribution from pre-control to post-control.

The cut-off of *L. loa* mf loads ≥20,000 mf/mL has been used as part of a pilot study on the efficacy of a test-and-not-treat (TNT) strategy in order to prevent not only the SAEs, but also to reduce the incidence of marked effects (with functional impairment for several days) which could have had an impact of the adherence of the population to the strategy [30]. We therefore estimated post-hoc the predicted prevalence of *L. loa* intensity ≥20,000 mf/mL (p20kmf). This was done by interpolation between the predicted prevalence of *L. loa* mf ≥10,000 (p10kmf) and the prevalence of *L. loa* mf ≥30,000 (p30kmf) using the average ratio (p20kmf-p30kmf)/(p10kmf-p30kmf) calculated from the data of the original RAPLOA study. The same method was also applied for the prediction of the prevalence of *L. loa* mf ≥8,000 mf/mL.

4.2 Impact of MDA on onchocerciasis

We used the mathematical model ONCHOSIM to predict how trends in *O. volvulus* mf prevalence would change over time since 1995 after implementation of mass treatment, for different pre-control endemicity levels and number of MDA treatment rounds for up to 30 years of MDA. ONCHOSIM is an established mathematical model for simulating transmission and control of onchocerciasis in a dynamic population [31]. It was developed in the 1990's and thereafter has been used extensively to support the decision-making of OCP in West-Africa and later also APOC [32–39]. The model assumes a systematic compliance model to ivermectin intake, and the exclusion of pregnant women and children below the age of five years old. Over the years, model predictions have been compared with various sorts of data, and the model has been shown to adequately mimic longitudinal trends in infection prevalence and intensity during mass treatment [40–42]. The model has been made publicly available and appropriate documentation has been published elsewhere [35].

A 200-year warm-up period was simulated before the impact of ivermectin was evaluated by ONCHOSIM in order to allow the human and worm populations to establish an endemic equilibrium[35], given pre-control transmission parameters. In absence of treatment, this equilibrium will continue to exist (e.g. onchocerciasis hypoendemic areas excluded from MDA). For areas that receive mass treatment, we simulated trends in village infection levels during MDA with ONCHOSIM as explained by Tekle *et al*. [43]. After each treatment round, the mean infection load is reduced. Various combinations of pre-control infection levels and annual treatment coverage levels were modelled in ONCHOSIM to predict trends in infection after annual treatment. The pre-control infection levels were expressed as community microfilarial load (CMFL), with CMFL 3, 5, 10, 20, 30, 50 and 70 mf/s, and assumed treatment coverage levels of 60%, 65%, 70%, 75% and 80%. For each pixel on the onchocerciasis map, the baseline prevalence was converted into a pre-control CMFL value, and values between the pre-set CMFL and treatment coverage values were interpolated between nearest higher and lower simulation scenarios to produce a draw of the pre-control *O. volvulus* mf prevalence (Pmf_0). As described, the changes in *O. volvulus* mf

prevalence were then applied at pixel-level for each epidemiological and programmatic situation (such as reported in Supplement S3), allowing us to generate raster maps of the predicted prevalence of *O. volvulus* infection post-control (Pmf_Y). The simulations used 5x5 km² raster maps for which we estimate for each 5x5 km² raster cell the predicted prevalence and the prediction standard error from the values predicted by the original 1x1 km² REMO/RAPLOA maps for the midpoint of the 5x5 km² raster cell. Infection levels were reported for 1995 (pre-control), 2015 and 2025.

4.3 Uncertainty analysis

Monte Carlo simulation with 500 iterations was used to propagate uncertainty in key inputs into our estimates of the prevalence of onchocerciasis, loiasis and onchocerciasis-loiasis co-infection with *L. loa* mf counts ≥20,000 mf/mL. Figure S5 summarizes the steps that were taken to generate samples of our key outputs for all 600,200 pixels on our map (corresponding to the number of pixels at 5x5 km² raster resolution). For onchocerciasis, this involved the following. First, rather than taking the point estimate of pre-control nodule prevalence from the REMO map, we sampled a random value from the normal distribution defined mean and its standard error, $N(P_{nod}, SE_{nod}^2)$. In the normal distribution, for each location and each run a random value for the nodule prevalence was generated from N (local predicted prevalence, local prediction standard error^2). Generated values outside (0,1) were set to 0 or 1. Next, given the sampled pre-control nodule prevalence, we generated a random value for the corresponding *O. volvulus* mf prevalence in the 5+ population based on a published functional relationship between the indicators mf prevalence in population aged 5+ and palpable nodules in males aged 20+.[44] Lastly, given the sampled pre-control *O. volvulus* mf prevalence and the local history of control, we used the stochastic microsimulation model ONCHOSIM to obtain a prediction of the expected *O. volvulus* mf prevalence in year Y. This was done by sampling a representative ONCHOSIM run from a large reference set of runs as explained above and elsewhere [43]. Briefly, this reference set of simulation runs contained the simulation output of 1,000 repeated simulation runs for each combination of a pre-defined set of pre-control infection levels (CMFL values, see above) and a set of treatment coverage levels (see above) [43]. Given the

sampled pre-control *O. volvulus* mf prevalence and the duration and average treatment coverage of MDA in the pixel of interest, we determined the four closest simulation scenarios (nearest simulated average pre-control mf prevalence below and above Pmf_0, and nearest simulated treatment coverage below and above the reported treatment coverage). We then sampled one run from each of the four closest simulations, extracting the model-predicted post-MDA *O. volvulus* mf prevalence for the time point of interest (determined by the duration of MDA) and calculated the predicted local *O. volvulus* mf prevalence through interpolation of the four ONCHOSIM predictions.

A similar procedure was followed for loiasis. First, we drew a random value from the normal distribution describing the uncertainty in the predicted prevalence of the RAPLOA map. Uncertainty regarding the conversion of the prevalence of history of eye worm into *L. loa* mf intensity distribution was propagated by sampling from the posterior distribution of parameter values of the statistical model described in section 3.44. Statistical uncertainty about the impact of a single ivermectin treatment on the intensity distribution of *L. loa* mf counts was propagated by sampling from the posterior distribution of transition probabilities, given the number of transitioning cases in the data and the assumption of a uniform Dirichlet prior for the transition probabilities.

The samples of the proportion of people in different *L. loa* mf intensity frequency classes generated and the *O. volvulus* infection prevalence were then combined to estimate the prevalence of co-infections of high *L. loa* mf intensity, as described in the manuscript, assuming that - within each pixel - the probability of an individual being mf-positive for *O. volvulus* was independent of the probability that they were mf-positive for *L. loa* (i.e. assuming no (non-spatial) autocorrelation between both filarial infections). This is in line with field data from areas where both filarial species overlap, and where high onchocerciasis and loiasis prevalence in communities does not necessarily imply co-infection with both diseases in that community [45]. Similarly, another study found some evidence of interaction between both species, but the immunological mechanisms are still not completely understood [46]. Therefore, we have not taken within-individual processes into account. Subsequently, the probability distribution per *L. loa* mf intensity class for both

O. volvulus endemic and non-endemic areas was multiplied by the total population size for 1995, 2015 and 2025.

The whole procedure was repeated 500 times for the main analysis to generate a posterior distribution of the mf prevalence of onchocerciasis and loiasis, and the prevalence of co-infection with *L. loa* mf counts ≥20,000 mf/mL. The resulting samples from the posterior distribution were summarised in terms of their means and 90%-Bayesian credible intervals (BCI). We calculated BCI for the prevalence of *L. loa* intensity of ≥20,000 mf/mL, using a prior, a vector of equal length as the categories with the prior information per category (non-zero positive number) and the number of Monte Carlo simulations to perform for construction of BCI. The percentiles we used to simulate were defaults to central 90%; 0.05, 0.95. The uncertainty analysis accounts for statistical random variation in the data, and although the BCI's per pixel are very wide as a result of the sequence of random processes that are simulated to predict the *L. loa* prevalences for each pixel, the combined results for thousands of pixels attenuates the effect of pixel-level deviations from the average value, resulting in narrow 90% BCIs (see Table 2, main manuscript). Uncertainties within the original data (e.g. methodologies applied in REMO or RAPLOA, choice of RAPLOA-surveyed areas, ONCHOSIM assumptions) are not taken into account in the uncertainty analysis. For the sensitivity analyses, the procedure was repeated 100 times.

Figure S5. Schematic presentation of the quantification of the uncertainty in the estimation of onchocerciasis-loiasis co-infection with *L. loa* mf counts of ≥20,000 per mL blood.

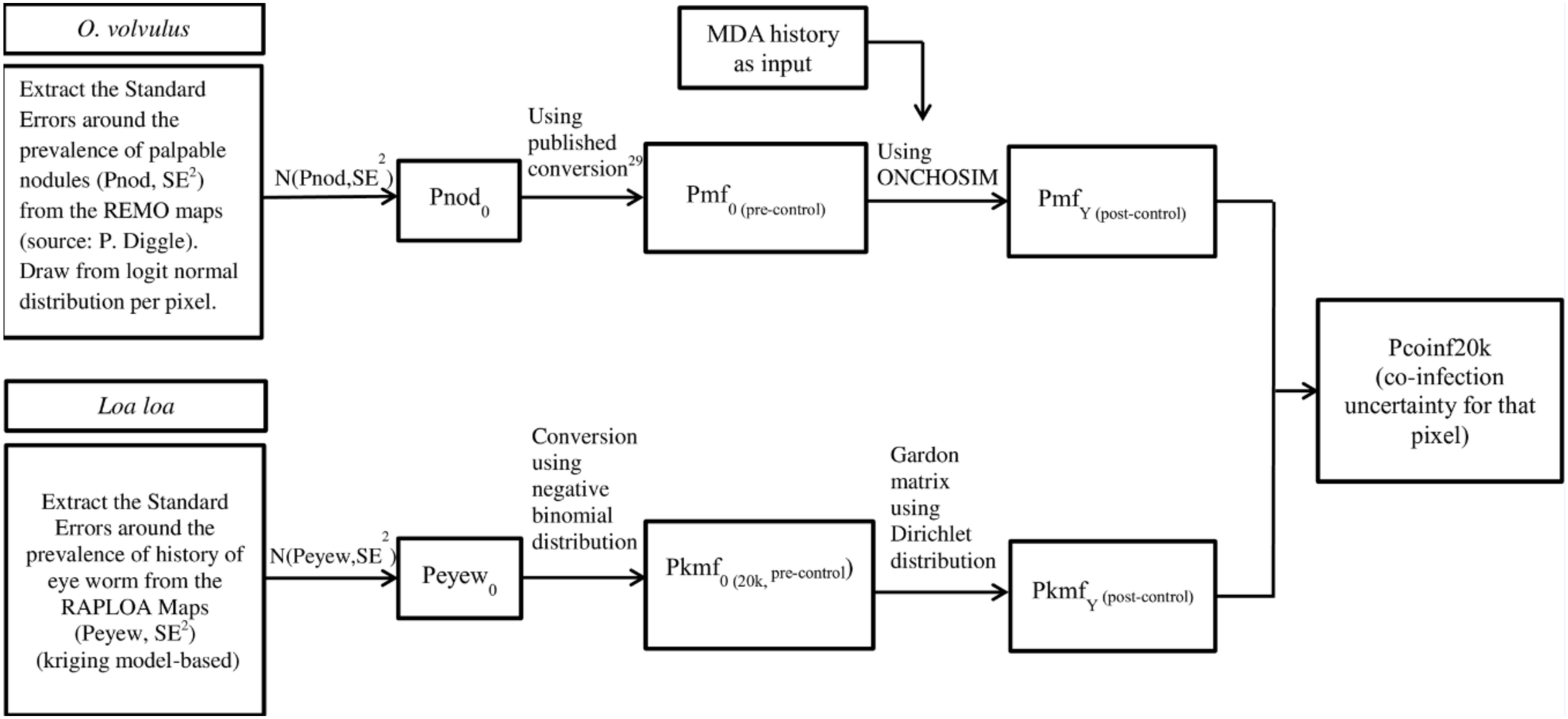

Note: We extracted the nodule prevalence (*Pnod*) at the pixel-level and the prediction standard errors (*SE*) given the *Pnod* at location *i*. We then translated the pre-control Pnod0 at each location *i* into a pre-control prevalence of *O. volvulus* mf in the general population aged 5+ (Pmf_0) using a previously published functional relationship. MDA history was then added as input for each location *i*, and ONCHOSIM was used to predict trends in prevalence of *O. volvulus* mf post-control (Pmf_Y) at time point *Y* (2015, 2025). Similarly, the prevalence of history of eye worm (Peyew) was extracted from the RAPLOA maps using kriging-based methods, with a standard error (*SE*) around the point estimation for each location *i*. The Peyew was then converted into the pre-control prevalence of *L. loa* mf for *L. loa* intensity of ≥k mf/mL ($Pkmf_0$; k=0, 1, 100, 500, 2000, 10000, 30000) using a negative binomial distribution (see above). In order to predict the post-control *L. loa* mf infection with ≥k mf/mL ($Pkmf_Y$) at time point *Y* (2015, 2025) based on the $Pkmf_0$ at a given location *i*, we used a Dirichlet distribution (see above). The Pmf_Y and each $Pkmf_Y$ were then multiplied with the population density at location i to estimate the local number of people with onchocerciasis infection and the number with *L. loa* infection intensities ≥k mf/mL The total number of people infected with onchocerciasis, the number with *L. loa,* the number with *L. loa* hypermicrofilaraemia (≥20,000 mf/mL) and the number of co-infected with onchocerciasis, was calculated by summation over all 600,200 pixels. This process was repeated 500 times to generate predictive distributions for each of these indicators with their 90% BCI.

5 Mathematical and statistical programmes

For the above described processes, we developed a computer programme in Python (Python 2.7) with the Numpy 1.7.1 library for matrix operations and the ARCPY site package to access ARCGIS 10.3 geoprocessing tools from within Python. These programmes converted the various raster maps into Numpy matrices, performed required calculations for each raster cell and aggregated the results into summary statistics at the specified levels. ArcGIS was used to spatially visualise the results.

6 References

1. APOC (1999) Guidelines for development of national plan and project proposal for sustainable community-directed treatment with ivermectin. *African Program Onchocerciasis Control;* APOC DOC.3.
2. Kim YE, Remme JHF, Steinmann P, Stolk WA, Roungou J-B, et al. (2015) Control, elimination, and eradication of river blindness: scenarios, timelines, and ivermectin treatment needs in Africa. *PLoS Negl Trop Dis* **9**: e0003664.
3. Habbema J, Stolk WA, Veerman JL, de Vlas SJ (2006) Report on a Health Impact Assessment of the African Programme for Onchocerciasis Control (APOC): "A rapid HIA." African Program Onchocerciasis Control.
4. Prost A, Hervouet J, Thylefors B (1979) The degrees of endemicity of onchocerciasis. *Bull World Heal Organ* **57**: 655–662.
5. Noma M, Nwoke B, Nutall I, Tambala P, Enyong P, et al. (n.d.) Rapid epidemiological mapping of onchocerciasis (REMO): its application by the African Programme for Onchocerciasis Control (APOC). *Ann Trop Med Parasitol 96 Suppl 1 S29-39*.
6. Thomson MC, Obsomer V, Kamgno J, Gardon J, Wanji S, et al. (2004) Mapping the distribution of Loa loa in Cameroon in support of the African Programme for Onchocerciasis Control. *Filaria J* **3**: 7.
7. Thomson MC, Obsomer V, Dunne M, Connor SJ, Molyneux DH (2000) Satellite mapping of Loa loa prevalence in relation to ivermectin use in west and central Africa. *Lancet* **356**: 1077–1078.
8. Wanji S, UNDP/World Bank/WHO Special Programme for Research and Training in Tropical Diseases (2001) Rapid Assessment Procedures for Loiasis: report of a multi-centre study. World Health Organization, Geneva, Switzerland.
9. Wanji S, Akotshi D, Mutro M, Tepage F, Ukety T, et al. (2012) Validation of the rapid assessment procedure for loiasis (RAPLOA) in the Democratic Republic of Congo. *Parasit Vectors* **5**.
10. Takougang I, Meremikwu M, Wandji S, Yenshu E V, Aripko B, et al. (2002) Rapid assessment method for prevalence and intensity of Loa loa infection. *Bull World Health Organ* **80**: 852–858.

11. Zouré HGM, Wanji S, Noma M, Amazigo UV, Diggle PJ, et al. (2011) The geographic distribution of Loa loa in Africa: results of large-scale implementation of the Rapid Assessment Procedure for Loiasis (RAPLOA). *PLoS Negl Trop Dis* **5**: e1210.

12. Diggle PJ, Thomson MC, Christensen OF, Rowlingson B, Obsomer V, et al. (2007) Spatial modelling and the prediction of Loa loa risk: decision making under uncertainty. *Ann Trop Med Parasitol* **101**: 499–509.

13. O'Hanlon SJ, Slater HC, Cheke RA, Boatin BA, Coffeng LE, et al. (2016) Model-Based Geostatistical Mapping of the Prevalence of Onchocerca volvulus in West Africa. *PLoS Negl Trop Dis* **10**: e0004328.

14. Noireau F, Nzoulani A, Sinda D, Itoua A (1990) Transmission indices of Loa loa in the Chaillu Mountains, Congo. *Am J Trop Med Hyg* **43**: 282–288.

15. Cano J, Basáñez M-G, O'Hanlon SJ, Tekle AH, Wanji S, et al. (2018) Identifying co-endemic areas for major filarial infections in sub-Saharan Africa: seeking synergies and preventing severe adverse events during mass drug administration campaigns. *Parasit Vectors* **11**: 70.

16. Kelly-Hope LA, Cano J, Stanton MC, Bockarie MJ, Molyneux DH (2014) Innovative tools for assessing risks for severe adverse events in areas of overlapping Loa loa and other filarial distributions: the application of micro-stratification mapping. *Parasit Vectors* **7**: 307.

17. Kelly-Hope LA, Unnasch TR, Stanton MC, Molyneux DH (2015) Hypo-endemic onchocerciasis hotspots: defining areas of high risk through micro-mapping and environmental delineation. *Infect Dis poverty* **4**: 36.

18. Zouré HG, Noma M, Tekle AH, Amazigo U V, Diggle PJ, et al. (2014) The geographic distribution of onchocerciasis in the 20 participating countries of the African Programme for Onchocerciasis Control: (2) pre-control endemicity levels and estimated number infected. *Parasit Vectors* **7**: 326.

19. Noma M, Zouré HG, Tekle AH, Enyong PA, Nwoke BE, et al. (2014) The geographic distribution of onchocerciasis in the 20 participating countries of the African Programme for Onchocerciasis Control: (1) priority areas for ivermectin treatment. *Parasit Vectors* **7**: 325.

20. APOC (2015) Guidelines for revising ivermectin treatment boundaries within the

context of onchocerciasis elimination. Annex to: "Report of the consultative meetings on strategic options and alternative treatment strategies for accelerating onchocerciasis elimination . African Program Onchocerciasis Control (APOC)/World Health Organization, **MG/15.21**.

21. Emukah E, Rakers LJ, Kahansim B, Miri ES, Nwoke BEB, et al. (2018) In southern Nigeria Loa loa blood microfilaria density is very low even in areas with high prevalence of loiasis: results of a survey using the new LoaScope technology. *Am Soc Trop Med Hyg* **99**: 116–123.
22. WHO-African Programme for Onchocerciasis Control (APOC) (2015) Report of the consultative meetings on strategic options and alternative treatment strategies for accelerating onchocerciasis elimination in Africa. *WHO/MG/1520*.
23. Mectizan-Taskforce (2004) Recommendations for the treatment of Onchocerciasis with Mectizan® in areas co-endemic for Onchocerciasis and Loiasis. *Mectizan® Expert Committee/Technical Consultative Committee*.
24. Herrador Z, Garcia B, Ncogo P, Perteguer MJ, Rubio JM, et al. (2018) Interruption of onchocerciasis transmission in Bioko Island: Accelerating the movement from control to elimination in Equatorial Guinea. *PLoS Negl Trop Dis* **12**: e0006471.
25. United Nations, Department of Economic and Social Affairs PD (2017). (n.d.) Population growth rates. World Population Prospects: The 2017 Revision, DVD Edition. Available online at: http://esa.un.org/ wpp/Excel-Data/population.htm. Accessed on 13 March 2018.
26. Harvest Choice (2015) Rural Population Density (pers./sq. km, 2000). International Food Policy Research Institute, Washington, DC., and University of Minnesota. Available online at: http://harvestchoice.org/data/pd00_rur). Accessed on 11 April 2017.
27. Gardon J, Kamgno J, Folefack G, Gardon-Wendel N, Bouchité B, et al. (1997) Marked decrease in Loa loa microfilaraemia six and twelve months after a single dose of ivermectin. *Trans R Soc Trop Med Hyg* **91**: 593–594.
28. Ranque S, Garcia A, Boussinesq M, Gardon J, Kamgno J, et al. (1996) Decreased prevalence and intensity of Loa loa infection in a community treated with ivermectin every three months for two years. *Trans R Soc Trop Med Hyg* **90**: 429–430.

29. Wanji S, Chounna Ndongmo WP, Fombad FF, Kengne-Ouafo JA, Njouendou AJ, et al. (2018) Impact of repeated annual community directed treatment with ivermectin on loiasis parasitological indicators in Cameroon: Implications for onchocerciasis and lymphatic filariasis elimination in areas co-endemic with Loa loa in Africa. *PLoS Negl Trop Dis* **12**: e0006750.

30. Kamgno J, Pion SD, Chesnais CB, Bakalar MH, D'Ambrosio M V, et al. (2017) A Test-and-Not-Treat strategy for onchocerciasis in Loa loa-endemic areas. *N Engl J Med* **377**: 2044–2052.

31. Plaisier A, van Oortmarssen G, Habbema J, Remme J, Alley E (1990) ONCHOSIM: a model and computer simulation program for the transmission and control of onchocerciasis. *Comput Methods Programs Biomed* **31**: 43–56.

32. Winnen M, Plaisier AP, Alley ES, Nagelkerke NJD, van Oortmarssen G, et al. (2002) Can ivermectin mass treatments eliminate onchocerciasis in Africa? *Bull World Health Organ* **80**: 384–391.

33. Alley WS, van Oortmarssen GJ, Boatin BA, Nagelkerke NJ, Plaisier AP, et al. (2001) Macrofilaricides and onchocerciasis control, mathematical modelling of the prospects for elimination. *BMC Public Health* **1**: 12.

34. Coffeng LE, Stolk WA, Zouré HGM, Veerman JL, Agblewonu KB, et al. (2014) African Programme for Onchocerciasis Control 1995–2015: Updated Health Impact Estimates Based on New Disability Weights. *PLoS Negl Trop Dis* **8**: e2759.

35. Stolk WA, Walker M, Coffeng LE, Basáñez M-G, de Vlas SJ (2015) Required duration of mass ivermectin treatment for onchocerciasis elimination in Africa: a comparative modelling analysis. *Parasit Vectors* **8**.

36. Coffeng LE, Stolk WA, Hoerauf A, Habbema D, Bakker R, et al. (2014) Elimination of African onchocerciasis: Modeling the impact of increasing the frequency of ivermectin mass treatment. *PLoS One* **9**: e115886.

37. Coffeng LE, Stolk WA, Zouré HGM, Veerman JL, Agblewonu KB, et al. (2013) African Programme For Onchocerciasis Control 1995-2015: model-estimated health impact and cost. *PLoS Negl Trop Dis* **7**: e2032.

38. Habbema J, Oostmarssen G, Plaisier A (1996) The ONCHOSIM model and its use in decision support for river blindness control. Cambridge Cambridge Univ Press *pp*

360–380.
39. Plaisier A (1996) Modelling onchocerciasis transmission and control [PhD Thesis]. *Rotterdam, Netherlands Erasmus Univ Rotterdam*.
40. Plaisier, A.P. et al. (1991) The risk and dynamics of onchocerciasis recrudescence after cessation of vector control. *Bull World Heal Organ* **69**: 169–178.
41. Plaisier AP, Alley ES, Boatin BA, Van Oortmarssen GJ, Remme H, et al. (1995) Irreversible effects of ivermectin on adult parasites in onchocerciasis patients in the Onchocerciasis Control Programme in West Africa. *J Infect Dis* **172**: 204–210.
42. Walker M, Stolk WA, Dixon MA, Bottomley C, Diawara L, et al. (2017) Modelling the elimination of river blindness using long-term epidemiological and programmatic data from Mali and Senegal. *Epidemics* **18**: 4–15.
43. Tekle A, Zouré H, Noma M, Boussinesq M, Coffeng LE, et al. (2016) Progress towards onchocerciasis elimination in the participating countries of the African Programme for Onchocerciasis Control: epidemiological evaluation results. *Infect Dis Poverty* **5**.
44. Coffeng LE, Pion SDS, O'Hanlon S, Cousens S, Abiose AO, et al. (2013) Onchocerciasis: the pre-control association between prevalence of palpable nodules and skin microfilariae. *PLoS Negl Trop Dis* **7**: e2168.
45. Ojurongbe O, Akindele AA, Adeleke MA, Oyedeji MO, Adedokun SA, et al. (2015) Co-endemicity of loiasis and onchocerciasis in rain forest communities in southwestern Nigeria. *PLoS Negl Trop Dis* **9**: e0003633.
46. Pion SDS, Clarke P, Filipe JAN, Kamgno J, Gardon J, et al. (2006) Co-infection with Onchocerca volvulus and Loa loa microfilariae in central Cameroon: are these two species interacting? *Parasitology* **132**: 843.
