# Supplementary material for: Projected Number of People With Onchocerciasis–Loiasis Coinfection in Africa, 1995 to 2025
Source: Clin Infect Dis. 2019 Jul 13;70(11):2281–9. doi: 10.1093/cid/ciz647 (PMC7245158; doi:10.1093/cid/ciz647)
Supplement: ciz647_suppl_Supplement_Information_S2 [file ciz647_suppl_supplement_information_s2.pdf]

Projected number of people with onchocerciasis-loiasis co-infection in Africa, 1995 to 2025

Supplementary information S2: Additional tables and figures

Natalie VS Vinkeles Melchers[1#], Luc E Coffeng[1], Michel Boussinesq[2], Belén Pedrique[3], Sébastien DS Pion[2], Afework H Tekle[4], Honorat GM Zouré[5], Samuel Wanji[6], Jan Remme[7*], Wilma A Stolk[1*]
* Contributed equally

[1] Department of Public Health, Erasmus MC, University Medical Center Rotterdam, P.O. box 2040, 3000 CA Rotterdam, The Netherlands
[2] Unité Mixte Internationale 233 « TransVIHMI », Institut de Recherche pour le Développement (IRD), INSERM U1175, University of Montpellier, Montpellier, France
[3] Drugs for Neglected Diseases *initiative*, 15 Chemin Louis Dunant, 1202 Geneva, Switzerland
[4] Preventive Chemotherapy and Transmission Control Unit, Control of Neglected Tropical Diseases Department, World Health Organization, Geneva, Switzerland
[5] ESPEN, World Health Organization, Regional Office for Africa, Cité du Djoué, Brazzaville, Republic of Congo
[6] Parasites and Vectors Research Unit, Department of Microbiology and Parasitology, University of Buea, Cameroon
[7] 120 Rue des Campanules, Ornex, France

*Correspondence to:
Natalie VS Vinkeles Melchers, Department of Public Health, Erasmus MC, University Medical Center Rotterdam, P.O. box 2040, 3000 CA Rotterdam, The Netherlands;
n.vinkelesmelchers@erasmusmc.nl, Natalie.melchers@gmail.com; +31 (0)10 70 38465

Table S1. Summary table for predicted *L. loa* mf-positive cases (any intensity) and hypermicrofilaraemia (≥20,000 mf/mL) in *L. loa*-mapped areas, without the presence of *O. volvulus* mf in the skin for 1995, 2015 and 2025. Absolute number of cases and population are presented in thousands.

Country	1995			2015			2025		
	Total *L. loa* mapped population at risk	Any intensity *L. loa* mf (% out of total *L. loa*-mapped population)	*L. loa* ≥20,000 mf/mL (% out of total *L. loa* mf cases)	Total *L. loa* mapped population at risk	Any intensity *L. loa* mf (% out of total *L. loa*-mapped population)	*L. loa* ≥20,000 mf/mL (% out of total *L. loa* mf cases)	Total *L. loa* mapped population at risk	Any intensity *L. loa* mf (% out of total *L. loa*-mapped population)	*L. loa* ≥20,000 mf/mL (% out of total *L. loa* mf cases)
Angola	5,158	156.5 (3.0%)	14.1 (9.0%)	9,224	283.2 (3.1%)	24.7 (8.7%)	11,681	402.3 (3.4%)	35.2 (8.7%)
Burundi	1,396	0.2 (0.01%)	0	2,104	0.1 (0.01%)	0	2,452	0.2 (0.01%)	0
Cameroon	6,829	822.7 (12.0%)	144.9 (17.6%)	10,624	1,084 (10.2%)	151.6 (14.0%)	12,823	1,351 (10.5%)	190.8 (14.1%)
CAR	1,866	405.1 (21.7%)	87.9 (21.7%)	2,711	553.6 (20.4%)	112.7 (20.3%)	3,258	724.7 (22.2%)	148.2 (20.4%)
Chad	3,681	52.3 (1.4%)	5.8 (11.0%)	6,660	62.4 (0.9%)	1.5 (2.3%)	8,511	80.6 (0.9%)	1.9 (2.3%)
Congo	1,789	256.1 (14.3%)	47.4 (18.5%)	2,931	387.8 (13.2%)	60.8 (15.7%)	3,612	511.1 (14.2%)	81.5 (15.9%)
DRC	28,849	1,288 (4.5%)	181.3 (14.1%)	48,821	1,730 (3.5%)	140.9 (8.1%)	61,575	2,102 (3.4%)	126.3 (6.0%)
Equatorial Guinea	209.4	61.6 (29.4%)	14.6 (23.7%)	376.1	115.5 (30.7%)	28.0 (24.2%)	468.6	162.5 (34.7%)	39.6 (24.4%)

Ethiopia	2,221	2.0 (0.1%)	0.1 (4.1%)	3,555	3.0 (0.1%)	0.1 (3.8%)	4,227	3.9 (0.1%)	0.2 (3.9%)
Gabon	261.8	80.9 (30.9%)	18.3 (22.6%)	397.0	126.8 (31.9%)	29.2 (23.0%)	474.1	166.5 (35.1%)	38.4 (23.1%)
Nigeria	23,966	477.1 (2.0%)	33.3 (7.0%)	38,928	559.2 (1.4%)	13.6 (2.4%)	49,524	735.2 (1.5%)	17.7 (2.4%)
South Sudan	3,826	84.7 (2.2%)	10.0 (11.9%)	6,188	94.6 (1.5%)	3.1 (3.2%)	7,634	118.7 (1.6%)	3.8 (3.2%)
Sudan	300.4	1.2 (0.4%)	0.1 (9.3%)	485.9	2.0 (0.4%)	0.2 (9.3%)	599.7	2.5 (0.4%)	0.2 (9.2%)
Uganda	979.3	10.1 (1.0%)	0.4 (3.8%)	1,819	14.7 (0.8%)	0.1 (0.7%)	2,419	20.9 (0.9%)	0.1 (0.6%)
**Grand total**	**81,331**	**3,698 (4.5%)**	**558.3 (15.1%)**	**134,823**	**5,017 (3.7%)**	**566.3 (11.3%)**	**169,257**	**6,382 (3.8%)**	**683.8 (10.7%)**

Table S2. Overall summary table with total predicted number of individuals positive for *O. volvulus* and *L. loa* mf with high intensity loiasis microfilaraemia (≥20,000 mf/mL) by pre-control endemicity and MDA initiation for 1995, 2015 and 2025. The percentages presented in the table between parentheses are the predicted number of cases with infection over the total population at risk for each category (row line). The first column is the predicted number of cases with onchocerciasis at baseline (1995); the second column is the predicted number of cases with onchocerciasis-loiasis co-infection and *L. loa* hypermicrofilaraemia at baseline. Subsequent predicted number of cases are also provided for 2015 and 2025. The classification of endemicity levels for each APOC-project is based on the published endemicity levels, provided by Kim *et al.* [1] and defined in table S1 of Supplement S1. This classification concerns locations with the specified endemicity level or below. Absolute number of cases are presented in thousands. Abbreviations: NA = Not applicable.

Pre-control endemicity level	Projections					
	1995		2015		2025	
	O. volvulus mf+	Co-infected with *L. loa* mf ≥20,000 mf/mL	*O. volvulus* mf+	Co-infected with *L. loa* mf ≥20,000 mf/mL	*O. volvulus* mf+	Co-infected with *L. loa* mf ≥20,000 mf/mL
**Hypoendemic**	**1,665 (18.7%)**	**14.9 (0.2%)**	**2,697 (18.4%)**	**23.7 (0.2%)**	**3,306 (18.1%)**	**28.9 (0.2%)**
<2010 MDA	24.4 (9.4%)	0	2.3 (0.5%)	0	<0.01 (<0.01%)	0
≥2010 - <2015 MDA	10.3 (19.3%)	0.1 (0.1%)	12.1 (12.7%)	0.01 (0.01%)	0.7 (0.6%)	0
≥2015 - <2025 MDA	18.8 (17.6%)	0.01 (0.01%)	31.7 (16.6%)	0.01 (<0.01%)	4.4 (1.8%)	0
No MDA	1,612 (19.0%)	14.9 (0.1%)	2,651 (19.0%)	23.7 (0.2%)	3,301 (19.0%)	28.9 (0.2%)

**Mesoendemic**	**2,115 (22.1%)**	**3.3 (0.03%)**	**888.8 (5.7%)**	**0.2 (<0.01%)**	**23.0 (0.1%)**	<0.01 (<0.01%)
<2010 MDA	1,850 (22.1%)	2.8 (0.03%)	580.7 (4.3%)	0.1 (<0.01%)	4.9 (0.03%)	<0.01 (<0.01%)
≥2010 - <2015 MDA	196.6 (26.1%)	0.4 (0.06%)	196.6 (15.7%)	0.1 (<0.01%)	4.1 (0.3%)	0
≥2015 - <2025 MDA	68.3 (15.7%)	0.03 (0.01%)	111.5 (14.9%)	0.02 (<0.01%)	13.9 (1.5%)	0
No MDA	NA	NA	NA	NA	NA	NA
**Hyperendemic**	**14,987 (37.4%)**	**119.0 (0.3%)**	**10,005 (15.1%)**	**34.0 (0.1%)**	**953.9 (1.2%)**	**2.2 (<0.01%)**
<2010 MDA	11,512 (37.1%)	76.0 (0.2%)	5,372 (10.5%)	5.2 (0.01%)	206.3 (0.3%)	0.4 (<0.01%)
≥2010 - <2015 MDA	2,568 (39.6%)	29.9 (0.5%)	3,134 (28.8%)	7.2 (0.1%)	348.3 (2.5%)	0.9 (0.01%)
≥2015 - <2025 MDA	907.0 (36.6%)	13.1 (0.5%)	1,498 (36.0%)	21.6 (0.5%)	399.4 (7.6%)	0.9 (0.02%)
No MDA	NA	NA	NA	NA	NA	NA
**Grand Total**	**18,767 (32.1%)**	**137.2 (0.2%)**	**13,591 (14.1%)**	**57.9 (0.1%)**	**4,283 (3.5%)**	**31.2 (0.03%)**

Table S3. Total predicted number of onchocerciasis-loiasis co-infected cases with *L. loa* hypermicrofilariaemia (≥20,000 mf/mL) by country, pre-control endemicity and MDA initiation for 1995, 2015 and 2025. These results presented here are according to our baseline assumption that ivermectin has an impact on loiasis prevalence and intensity after one round of annual MDA, whereas further treatment assists in sustaining the *L. loa* mf count distribution but does not induce further changes. The classification of endemicity levels for each APOC-project is based on the published endemicity levels provided by Kim *et al.* [1] and defined in table S1 of Supplement S1. This classification concerns locations with the specified endemicity level or below. Absolute number of cases and population are presented in thousands.

**1995**			
**Country**	**Total population at risk**	***O. volvulus* infected cases**	**Co-infected cases with *L. loa* ≥20,000 mf/mL**
**Angola**			
**Hypo**	274.9	45.6	0.1
**Meso**	787.3	170.9	0.2
**Hyper**	64.3	13.1	<0.01
**Burundi**			
**Hypo**	0.0	0.0	0.0
**Meso**	582.8	171.5	<0.01
**Hyper**	NA	NA	NA
**Cameroon**			
**Hypo**	1,012	182.1	7.8
**Meso**	NA	NA	NA
**Hyper**	4,452	2,109	26.9

**Central African Republic (CAR)**			
**Hypo**	83.5	17.2	1.0
**Meso**	NA	NA	NA
**Hyper**	1,016	425.2	8.5
**Chad**			
**Hypo**	0.0	0.0	0.0
**Meso**	NA	NA	NA
**Hyper**	1,144	344.9	1.5
**Congo**			
**Hypo**	327.5	54.7	1.5
**Meso**	NA	NA	NA
**Hyper**	580.1	152.4	3.0
**Democratic Republic of Congo (DRC)**			
**Hypo**	3,994	753.7	3.3
**Meso**	280.5	32.7	0.01
**Hyper**	18,996	7,478	69.9
**Equatorial Guinea**			
**Hypo**	0.0	0.0	0.0
**Meso**	NA	NA	NA
**Hyper**	41.8	0.0	0.0
**Ethiopia**			
**Hypo**	775.9	206.1	<0.01
**Meso**	267.6	78.3	<0.01
**Hyper**	1,102	486.4	0.01
**Gabon**			
**Hypo**	55.0	9.3	0.7
**Meso**	NA	NA	NA
**Hyper**	NA	NA	NA
**Nigeria**			
**Hypo**	2,158	370.4	0.4
**Meso**	7,650	1,662	3.1

**Hyper**	9,610	3,063	6.0
**South Sudan**			
**Hypo**	NA	NA	NA
**Meso**	NA	NA	NA
**Hyper**	2,406	692.5	2.9
**Sudan**			
**Hypo**	167.3	23.4	0.02
**Meso**	NA	NA	NA
**Hyper**	NA	NA	NA
**Uganda**			
**Hypo**	44.4	2.5	<0.01
**Meso**	NA	NA	NA
**Hyper**	609.9	223.0	0.12
**Grand Total**	**58,484**	**18,767**	**137.2**

`

**2015**												
	**MDA initiation**											
**Country**	**<2010**			**≥2010 - <2015**			**≥2015 - <2025**			**No MDA planned**		
	**Total population at risk**	***O. volvulus* infected cases**	**Co-infected cases with *L. loa* ≥20,000 mf/mL**	**Total population at risk**	***O. volvulus* infected cases**	**Co-infected cases with *L. loa* ≥20,000 mf/mL**	**Total population at risk**	***O. volvulus* infected cases**	**Co-infected cases with *L. loa* ≥20,000 mf/mL**	**Total population at risk**	***O. volvulus* infected cases**	**Co-infected cases with *L. loa* ≥20,000 mf/mL**
**Angola**												
**Hypo**	NA	NA	NA	95.3	12.1	0.01	190.8	31.7	0.01	204.8	29.4	0.1
**Meso**	816.5	69.7	0.01	340.1	51.8	<0.01	249.4	55.7	0.01	NA	NA	NA
**Hyper**	NA	NA	NA	NA	NA	NA	114.8	22.1	<0.01	NA	NA	NA
**Burundi**												
**Hypo**	NA	NA	NA	NA	NA	NA	NA	NA	NA	0.0	0.0	0.0
**Meso**	877.8	33.3	<0.01	NA	NA	NA	NA	NA	NA	NA	NA	NA
**Hyper**	NA	NA	NA	NA	NA	NA	NA	NA	NA	NA	NA	NA
**Cameroon**												
**Hypo**	NA	NA	NA	NA	NA	NA	NA	NA	NA	1,574	283.4	12.2
**Meso**	NA	NA	NA	NA	NA	NA	NA	NA	NA	NA	NA	NA
**Hyper**	6,772	542.9	1.2	NA	NA	NA	152.6	62.1	1.87	NA	NA	NA
**Central African Republic (CAR)**												
**Hypo**	NA	NA	NA	NA	NA	NA	NA	NA	NA	121.4	24.9	1.5
**Meso**	NA	NA	NA	NA	NA	NA	NA	NA	NA	NA	NA	NA
**Hyper**	1,428	16.1	0.03	NA	NA	NA	48.3	19.7	1.1	NA	NA	NA
**Chad**												
**Hypo**	NA	NA	NA	NA	NA	NA	NA	NA	NA	0.0	0.0	0.0
**Meso**	NA	NA	NA	NA	NA	NA	NA	NA	NA	NA	NA	NA
**Hyper**	2,069	4.5	<0.01	NA	NA	NA	NA	NA	NA	NA	NA	NA

**Congo**												
**Hypo**	NA	NA	NA	NA	NA	NA	NA	NA	NA	536.5	89.5	2.4
**Meso**	NA	NA	NA	NA	NA	NA	NA	NA	NA	NA	NA	NA
**Hyper**	908.4	86.1	0.2	41.8	21.8	0.04	NA	NA	NA	NA	NA	NA
**Democratic Republic of Congo (DRC)**												
**Hypo**	NA	NA	NA	NA	NA	NA	NA	NA	NA	6,757	1,276	5.6
**Meso**	NA	NA	NA	5.7	2.7	0.0	468.9	49.5	0.01	NA	NA	NA
**Hyper**	20,232	3,725	3.3	8,480	2,717	7.1	3,426	1,238	18.6	NA	NA	NA
**Equatorial Guinea**												
**Hypo**	NA	NA	NA	NA	NA	NA	NA	NA	NA	0.0	0.0	0.0
**Meso**	NA	NA	NA	NA	NA	NA	NA	NA	NA	NA	NA	NA
**Hyper**	74.8	0.0	0.0	NA	NA	NA	NA	NA	NA	NA	NA	NA
**Ethiopia**												
**Hypo**	NA	NA	NA	0	313.0	1,257	NA	NA	NA	1,242	330.0	<0.01
**Meso**	0	14.1	398.0	0	6.6	32.6	NA	NA	NA	NA	NA	NA
**Hyper**	1,590	100.7	<0.01	NA	NA	NA	173.3	60.8	<0.01	NA	NA	NA
**Gabon**												
**Hypo**	NA	NA	NA	NA	NA	NA	NA	NA	NA	83.37	14.14	1.1
**Meso**	NA	NA	NA	NA	NA	NA	NA	NA	NA	NA	NA	NA
**Hyper**	NA	NA	NA	NA	NA	NA	NA	NA	NA	NA	NA	NA
**Nigeria**												
**Hypo**	299.1	1.8	0.0	NA	NA	NA	NA	NA	NA	3,206	569.0	0.7
**Meso**	11,518	462.0	0.1	907.6	142.1	0.06	NA	NA	NA	NA	NA	NA
**Hyper**	15,391	584.5	0.1	NA	NA	NA	218.1	84.4	0.05	NA	NA	NA
**South Sudan**												
**Hypo**	NA	NA	NA	NA	NA	NA	NA	NA	NA	NA	NA	NA
**Meso**	NA	NA	NA	NA	NA	NA	NA	NA	NA	NA	NA	NA
**Hyper**	1,740	269.6	0.3	2,121	380.2	0.03	28.7	10.2	0.03	NA	NA	NA
**Sudan**												

**Hypo**	50.9	0.5	0.0	NA	NA	NA	NA	NA	NA	219.5	35.1	0.03
**Meso**	NA	NA	NA	NA	NA	NA	NA	NA	NA	NA	NA	NA
**Hyper**	NA	NA	NA	NA	NA	NA	NA	NA	NA	NA	NA	NA
**Uganda**												
**Hypo**	82.5	0.0	0.0	NA	NA	NA	NA	NA	NA	NA	NA	NA
**Meso**	NA	NA	NA	NA	NA	NA	NA	NA	NA	NA	NA	NA
**Hyper**	898.4	43.2	<0.01	234.0	14.9	<0.01	NA	NA	NA	NA	NA	NA
**Grand total**	**65,145**	**5,956**	**5.3**	**12,226**	**3,343**	**7.3**	**5,102**	**1,641**	**21.6**	**13,945**	**2,651**	**23.7**

Country	<2010			≥2010 - <2015			≥2015 - <2025			No MDA planned		
	**2025**											
	**MDA initiation**											
	**Total population at risk**	***O. volvulus* infected cases**	**Co-infected cases with *L. loa* ≥20,000 mf/mL**	**Total population at risk**	***O. volvulus* infected cases**	**Co-infected cases with *L. loa* ≥20,000 mf/mL**	**Total population at risk**	***O. volvulus* infected cases**	**Co-infected cases with *L. loa* ≥20,000 mf/mL**	**Total population at risk**	***O. volvulus* infected cases**	**Co-infected cases with *L. loa* ≥20,000 mf/mL**
**Angola**												
**Hypo**	NA	NA	NA	120.2	0.7	<0.01	240.8	4.4	<0.01	259	37.0	0.1
**Meso**	1,030	1.6	<0.01	429.3	2.4	0.0	314.8	8.8	<0.01	NA	NA	NA
**Hyper**	NA	NA	NA	NA	NA	NA	144.8	3.5	0.0	NA	NA	NA
**Burundi**												
**Hypo**	NA	NA	NA	NA	NA	NA	NA	NA	NA	NA	NA	NA
**Meso**	1,019	0.1	0.0	NA	NA	NA	NA	NA	NA	NA	NA	NA
**Hyper**	NA	NA	NA	NA	NA	NA	NA	NA	NA	NA	NA	NA
**Cameroon**												
**Hypo**	NA	NA	NA	NA	NA	NA	NA	NA	NA	1,899	341.5	14.6
**Meso**	NA	NA	NA	NA	NA	NA	NA	NA	NA	NA	NA	NA
**Hyper**	8,169	26.4	0.1	NA	NA	NA	184.1	9.3	0.04	NA	NA	NA
**Central African Republic (CAR)**												
**Hypo**	NA	NA	NA	NA	NA	NA	NA	NA	NA	146	29.8	1.8
**Meso**	NA	NA	NA	NA	NA	NA	NA	NA	NA	NA	NA	NA
**Hyper**	1,714	0.1	<0.01	NA	NA	NA	58.0	4.8	0.04	NA	NA	NA
**Chad**												
**Hypo**	NA	NA	NA	NA	NA	NA	NA	NA	NA	NA	NA	NA
**Meso**	NA	NA	NA	NA	NA	NA	NA	NA	NA	NA	NA	NA
**Hyper**	2,641	<0.01	0.0	NA	NA	NA	NA	NA	NA	NA	NA	NA

**Congo**												
**Hypo**	NA	NA	NA	NA	NA	NA	NA	NA	NA	661	110.1	3.0
**Meso**	NA	NA	NA	NA	NA	NA	NA	NA	NA	NA	NA	NA
**Hyper**	1,119	3.0	0.01	51.5	2.5	<0.01	NA	NA	NA	NA	NA	NA
**Democratic Republic of Congo (DRC)**												
**Hypo**	NA	NA	NA	NA	NA	NA	NA	NA	NA	8,516	1,607	7.1
**Meso**	NA	NA	NA	7.1	0.4	0.0	591.0	4.9	<0.01	NA	NA	NA
**Hyper**	25,500	154.4	0.3	10,689	320.2	0.9	4,318	369.0	0.9	NA	NA	NA
**Equatorial Guinea**												
**Hypo**	NA	NA	NA	NA	NA	NA	NA	NA	NA	NA	NA	NA
**Meso**	NA	NA	NA	NA	NA	NA	NA	NA	NA	NA	NA	NA
**Hyper**	92.8	0.0	0.0	NA	NA	NA	NA	NA	NA	NA	NA	NA
**Ethiopia**												
**Hypo**	NA	NA	NA	NA	NA	NA	NA	NA	NA	1,477	391.8	<0.01
**Meso**	472.4	0.1	0.0	NA	NA	NA	36.9	0.3	0.0	NA	NA	NA
**Hyper**	1,890	0.4	0.0	NA	NA	NA	206.1	4.4	0.0	NA	NA	NA
**Gabon**												
**Hypo**	NA	NA	NA	NA	NA	NA	NA	NA	NA	99.3	16.8	1.3
**Meso**	NA	NA	NA	NA	NA	NA	NA	NA	NA	NA	NA	NA
**Hyper**	NA	NA	NA	NA	NA	NA	NA	NA	NA	NA	NA	NA
**Nigeria**												
**Hypo**	380.4	<0.01	0.0	NA	NA	NA	NA	NA	NA	4,077	724.0	0.9
**Meso**	14,647	3.2	<0.01	1,154	1.4	<0.01	NA	NA	NA	NA	NA	NA
**Hyper**	19,573	10.2	<0.01	NA	NA	NA	277.4	6.3	<0.01	NA	NA	NA
**South Sudan**												
**Hypo**	NA	NA	NA	NA	NA	NA	NA	NA	NA	NA	NA	NA
**Meso**	NA	NA	NA	NA	NA	NA	NA	NA	NA	NA	NA	NA
**Hyper**	2,144	11.1	0.01	2,613	25.3	<0.01	35.4	2.0	<0.01	NA	NA	NA
**Sudan**												

**Hypo**	62.7	<0.01	0.0	NA	NA	NA	NA	NA	NA	270	43.2	0.03
**Meso**	NA	NA	NA	NA	NA	NA	NA	NA	NA	NA	NA	NA
**Hyper**	NA	NA	NA	NA	NA	NA	NA	NA	NA	NA	NA	NA
**Uganda**												
**Hypo**	109.6	0.0	0.0	NA	NA	NA	NA	NA	NA	NA	NA	NA
**Meso**	NA	NA	NA	NA	NA	NA	NA	NA	NA	NA	NA	NA
**Hyper**	1,193	0.6	<0.01	310.9	0.3	0.0	NA	NA	NA	NA	NA	NA
**Grand Total**	**81,759**	**211.2**	**0.4**	**15,375**	**353.2**	**0.9**	**6,407**	**417.7**	**1.0**	**17,404**	**3,302**	**28.9**

Table S4. Overview of the projections for number of *O. volvulus*, *L. loa* and co-infected cases for 1995, 2015 and 2025. The percentages between parentheses in each row are based on the total number of people living in the respective areas. Results of the sensitivity analyses of the impact of ivermectin on *L. loa* prevalence and intensity are presented by year in the different column panels. Absolute number of cases and population are presented in thousands. Abbreviations: IVM = ivermectin; MDA = Mass Drug Administration with ivermectin.

	1995	2015			2025		
	Pre-control	No effect of IVM	Effect 1st Tx only	Exponential effect	No effect of IVM	Effect 1st Tx only	Exponential effect
**I. Total number of cases in *L. loa* mapped areas**							
Total no. of people	81,331	134,823			169,257		
No. (%) of people with any *L. loa* mf intensity	3,698 (4.5%)	6,218 (4.6%)	5,017 (3.7%)	4,724 (3.5%)	7,724 (4.6%)	6,382 (3.8%)	5,198 (3.1%)
No. (%) of people with *L. loa* hypermicrofilariamia	558.3 (0.7%)	707.6 (0.5%)	566.3 (0.4%)	393.1 (0.3%)	872.7 (0.5%)	683.8 (0.4%)	420.0 (0.2%)
**II. Total number of cases in *L. loa*-mapped areas that are endemic for onchocerciasis** *(sum of areas where MDA is not applied and areas where MDA is applied)*							
**Areas where MDA is applied**							
Total no. of people	50,011	82,472			103,541		
No. (%) of people with *O. volvulus* mf	17,156 (34.3%)	10,940 (13.3%)			982.0 (0.9%)		
No. (%) of people with any *L. loa* mf intensity	2,093 (4.2%)	3,436 (4.2%)	2,356 (2.9%)	625.7 (0.8%)	4,284 (4.1%)	2,799 (2.7%)	8.8 (0.01%)

No. (%) of people with *L. loa* hypermicrofilariamia	287.5 (0.6%)	471.8 (0.6%)	118.5 (0.1%)	62.6 (0.1%)	587.1 (0.6%)	83.9 (0.08%)	<0.01 (<0.01%)
No. (%) of co-infected cases with any *L. loa* mf intensity	865.7 (1.7%)	661.6 (0.8%)	484.6 (0.6%)	233.1 (0.3%)	95.3 (0.1%)	64.1 (0.06%)	0.8 (<0.01%)
No. (%) of co-infected cases with *L. loa* hypermicrofilariamia	122.3 (0.2%)	98.7 (0.1%)	34.2 (0.04%)	24.6 (0.03%)	15.4 (0.01%)	2.2 (0.002%)	<0.01 (<0.01%)
**Areas where MDA is not applied (hypoendemic for onchocerciasis; MDA contraindicated according to MEC/TCC guidelines because of suspected loaisis co-endemicity)**							
Total no. of people	8,473	13,945			17,404		
No. (%) of people with *O. volvulus* mf	1,612 (19.0%)	2,651 (19.0%)			3,302 (19.0%)		
No. (%) of people with any *L. loa* mf intensity	508.2 (6.0%)	815.4 (5.8%)	815.7 (5.8%)	816.0 (5.9%)	1,004 (5.8%)	1,004 (5.8%)	1,005 (5.8%)
No. (%) of people with *L. loa* hypermicrofilariamia	81.1 (1.0%)	128.9 (0.9%)	128.9 (0.9%)	129.0 (0.9%)	157.8 (0.9%)	157.8 (0.9%)	158.0 (0.9%)
No. (%) of co-infected cases with any *L. loa* mf intensity	93.2 (1.1%)	149.6 (1.1%)	149.7 (1.1%)	149.7 (1.1%)	183.9 (1.1%)	183.9 (1.1%)	184.1 (1.1%)
No. (%) of co-infected cases with *L. loa* hypermicrofilariamia	14.9 (0.2%)	23.7 (0.2%)	23.7 (0.2%)	23.7 (0.2%)	28.9 (0.2%)	28.9 (0.2%)	28.9 (0.2%)

References

1. Kim YE, Remme JHF, Steinmann P, Stolk WA, Roungou J-B, et al. (2015) Control, elimination, and eradication of river blindness: scenarios, timelines, and ivermectin treatment needs in Africa. *PLoS Negl Trop Dis* **9**: e0003664.
