# Supplementary material for: Projected Number of People With Onchocerciasis–Loiasis Coinfection in Africa, 1995 to 2025
Source: Clin Infect Dis. 2019 Jul 13;70(11):2281–9. doi: 10.1093/cid/ciz647 (PMC7245158; doi:10.1093/cid/ciz647)
Supplement: ciz647_suppl_Supplement_Information_S3 [file ciz647_suppl_supplement_information_s3.pdf]

Projected number of people with onchocerciasis-loiasis co-infection in Africa, 1995 to 2025

Supplementary information S3:

Treatment history and assumptions per APOC project

Natalie VS Vinkeles Melchers[1#], Luc E Coffeng[1], Michel Boussinesq[2], Belén Pedrique[3], Sébastien DS Pion[2], Afework H Tekle[4], Honorat GM Zouré[5], Samuel Wanji[6], Jan Remme[7*], Wilma A Stolk[1*]
* Contributed equally

[1] Department of Public Health, Erasmus MC, University Medical Center Rotterdam, P.O. box 2040, 3000 CA Rotterdam, The Netherlands.
[2] Unité Mixte Internationale 233 « TransVIHMI », Institut de Recherche pour le Développement (IRD), INSERM U1175, University of Montpellier, Montpellier, France.
[3] Drugs for Neglected Diseases *initiative*, 15 Chemin Louis Dunant, 1202 Geneva, Switzerland.
[4] Preventive Chemotherapy and Transmission Control Unit, Control of Neglected Tropical Diseases Department, World Health Organization, Geneva, Switzerland.
[5] ESPEN, World Health Organization, Regional Office for Africa, Cité du Djoué, Brazzaville, Republic of Congo.
[6] Parasites and Vectors Research Unit, Department of Microbiology and Parasitology, University of Buea, Cameroon.
[7] 120 Rue des Campanules, Ornex, France.

*Correspondance to:
Natalie VS Vinkeles Melchers, Department of Public Health, Erasmus MC, University Medical Center Rotterdam, P.O. box 2040, 3000 CA Rotterdam, the Netherlands n.vinkelesmelchers@erasmusmc.nl, Natalie.melchers@gmail.com;
+31 (0)10 70 38465

Table S1. Table with estimated population at risk, pre-control endemicity level, treatment history and future treatment scenarios based on assumptions, per country and APOC-project.

Country	APOC project (MDA implementation unit)	Estimate of population living in RAPLOA-surveyed area in 2015	Assumed pre-control endemicity level[1]	Reported or assumed year of MDA initiation[2]	Reported or assumed MDA coverage[2] in %	MDA frequency per annum
Angola	Bengo	26,740	Mesoendemic	2010	74.9	1
Angola	Benguela	42,137	Mesoendemic	2012	62.7	1
Angola	Cuanza Norte	26,393	Mesoendemic	2011	74.9	1
Angola	Huila	180,223	Mesoendemic	2010	69.9	1
Angola	Kuando Kubango	242,809	Mesoendemic	2009	76.4	1
Angola	Lunda Norte	313,811	Hyperendemic	2009	70.9	1
Angola	Lunda sul	259,844	Mesoendemic	2009	68.9	1
Angola	Moxico 1	159,908	Hyperendemic	2011	68.3	1
Angola	Namibe	35,283	Mesoendemic	2015	75.0	1
Angola	NY Benguela	114,754	Hyperendemic	2015	65.0	1
Angola	NY Cuanza Norte	19,275	Mesoendemic	2015	62.7	1
Angola	NY Huila	22,823	Mesoendemic	2015	65.0	1
Angola	NY Lunda Norte	68,105	Mesoendemic	2015	65.0	1
Angola	NY Moxico 1	88,947	Mesoendemic	2015	65.0	1
Angola	P5Angola	204,828	Hypoendemic	No Tx	-	-
Angola	Uige	191,298	Hyperendemic	2015	62.7	1
Angola	Zaire	14,469	Mesoendemic	2015	62.7	1
Burundi	Bururi	8,310	Mesoendemic	2008	75.3	1
Burundi	Cibitoke-Bubanza	869,440	Mesoendemic	2006	79.0	1
Burundi	P5Burundi	194,746	Hypoendemic*	No Tx	-	-
Cameroon	Adamaoua 1	506,019	Hyperendemic	2008	74.4	1
Cameroon	Adamaoua 2	460,959	Hyperendemic	2004	77.4	1
Cameroon	Centre 1	456,977	Hyperendemic	2005	60.0	1

Cameroon	Centre 2	110,827	Hyperendemic	2005	76.6	1
Cameroon	Centre 3	353,235	Hyperendemic	2004	77.4	1
Cameroon	East	115,730	Hyperendemic	2007	82.7	1
Cameroon	Littoral 1	307,714	Hyperendemic	2007	68.3	1
Cameroon	Littoral 2	162,192	Hyperendemic	2006	60.0	1
Cameroon	Northern	573,384	Hyperendemic	2004	65.0	1
Cameroon	Northwest	890,910	Hyperendemic	2005	78.2	1
Cameroon	P20Cameroon	152,583	Hyperendemic	2015	78.3	1
Cameroon	P5Cameroon	1,574,095	Hypoendemic	No Tx	-	-
Cameroon	South	319,404	Hyperendemic	2006	77.1	1
Cameroon	South West 1	426,951	Hyperendemic	2005	80.9	1
Cameroon	South West 2	278,034	Hyperendemic	2004	83.7	1
Cameroon	Western	1,809,284	Hyperendemic	2003	80.0	1
CAR	CAR region 3	424,325	Hyperendemic	1996	75.0	1
CAR	CAR region 4	379,742	Hyperendemic	1997	75.0	1
CAR	CAR region 5	187,932	Hyperendemic	1998	75.0	1
CAR	CAR region 6	435,918	Hyperendemic	2003	75.0	1
CAR	P20CAR	48,295	Hyperendemic	2016	75.0	1
CAR	P5CAR	121,387	Hypoendemic	No Tx	-	-
Chad	Chad	2,068,602	Hyperendemic	2001	81.0	1
Chad	P5 Chad propext	123,090	Hypoendemic*	No Tx	-	-
Chad	P5Chad	211,790	Hypoendemic*	No Tx	-	-
Congo	Congo 1	908,363	Hyperendemic	2008	65.0	1
Congo	P20Congo	41,827	Hyperendemic	2014	81.1	1
Congo	P5Congo	536,489	Hypoendemic	No Tx	-	-
DRC	Bandundu	3,668	Hyperendemic	2005	82.1	1
DRC	Bas-Congo Kinshasa	1,540,019	Hyperendemic	2008	70.1	1
DRC	Butembo-Beni	863,124	Hyperendemic	2011	69.3	1
DRC	Equateur-Kiri	1,261,084	Hyperendemic	2009	78.8	1
DRC	Ituri-Nord	1,160,699	Hyperendemic	2009	81.2	1
DRC	Ituri-Sud	1,136,715	Hyperendemic	2012	71.9	1
DRC	Kasai	9,540,955	Hyperendemic	2009	75.3	1
DRC	Kasongo	1,219,561	Hyperendemic	2009	70.7	1
DRC	Katanga-Nord	646,562	Hyperendemic	2009	79.8	1

DRC	Katanga-Sud	459,476	Hyperendemic	2009	76.1	1
DRC	Lualaba	234,079	Hyperendemic	2008	80.4	1
DRC	Lubutu	293,679	Hyperendemic	2009	73.7	1
DRC	Masisi-Walikale	1,029,085	Hyperendemic	2010	73.1	1
DRC	Mongala	1,448,760	Hyperendemic	2009	78.1	1
DRC	NY Katanga-Nord	468,923	Mesoendemic	2015	71.9	1
DRC	NY Lualaba	1,017,774	Hyperendemic	2015	71.9	1
DRC	NY Masisi-Walikale	44,320	Hyperendemic	2015	71.9	1
DRC	NY Rutshuru-Ngoma	5,673	Mesoendemic	2014	71.9	1
DRC	NY Sankuru	481,326	Hyperendemic	2014	71.9	1
DRC	NY Ueles	166,371	Hyperendemic	2014	71.9	1
DRC	P20DRC	2,363,880	Hyperendemic	2016	71.9	1
DRC	P5DRC	6,756,957	Hypoendemic	No Tx	-	-
DRC	Rutshuru-Ngoma	218,493	Hyperendemic	2009	74.0	1
DRC	Sankuru	1,071,797	Hyperendemic	2007	65.0	1
DRC	Tshopo	1,366,088	Hyperendemic	2010	67.5	1
DRC	Tshuapa	1,216,233	Hyperendemic	2010	66.2	1
DRC	Ubangi-Nord	824,908	Hyperendemic	2011	69.0	1
DRC	Ubangi-Sud	1,396,619	Hyperendemic	2011	72.2	1
DRC	Ueles	1,133,182	Hyperendemic	2006	65.0	1
Equatorial Guinea	Bioko	74,833	Hyperendemic	1990 – 2016[3]	71.0	1
Equatorial Guinea	P5EqGuinea	285,591	Hypoendemic*	No Tx	-	-
Ethiopia	Assosa	11,693	Mesoendemic	2015	80.0	2
Ethiopia	Bench-Maji	374,603	Hyperendemic	2005	76.3	1
Ethiopia	Gambella	32,009	Hyperendemic	2006	77.5	1
Ethiopia	Illubabor	114,184	Hyperendemic	2004	81.8	1
Ethiopia	Kaffa-Sheka	245,823	Hyperendemic	2003	78.5	1
Ethiopia	Kamashi	51,814	Hyperendemic	2015	80.0	2
Ethiopia	Metekel	148,463	Mesoendemic	2007	74.1	1
Ethiopia	North Gondar	248,860	Mesoendemic	2004	74.4	1
Ethiopia	NY West Wellega	19,337	Mesoendemic	2015	80.0	2
Ethiopia	P20Ethiopia	121,525	Hyperendemic	2015	80.0	2
Ethiopia	P5Ethiopia	1,241,987	Hypoendemic	No Tx	-	2
Ethiopia	West Wellega	823,279	Hyperendemic	2006	81.7	1

Gabon	P5Gabon	83,371	Hypoendemic	No Tx	-	-
Nigeria	Adamawa	237,052	Hyperendemic	2001	79.8	1
Nigeria	Akwa Ibom	29,808	Mesoendemic	2006	83.7	1
Nigeria	Bauchi	1,672,080	Mesoendemic	2009	78.7	1
Nigeria	Benue	3,792,809	Hyperendemic	2007	76.7	1
Nigeria	Borno	33,912	Mesoendemic	2006	83.2	1
Nigeria	Cross River	1,118,775	Hyperendemic	2003	60.0	1
Nigeria	Edo Delta	865,175	Hyperendemic	2006	65.0	1
Nigeria	Ekiti	2,380,941	Mesoendemic	2007	76.3	1
Nigeria	Enugu Anambra Ebony	2,226,668	Hyperendemic	1999	80.3	1
Nigeria	FCT	381,667	Mesoendemic	2004	81.9	1
Nigeria	Gombe	1,104,667	Hyperendemic	2006	82.1	1
Nigeria	Imo Abia	1,216,750	Hyperendemic	1999	78.0	1
Nigeria	Jigawa	256,449	Hypoendemic	2004	73.9	1
Nigeria	Kaduna	669,034	Mesoendemic	1992	79.8	1
Nigeria	Kano	242,115	Hyperendemic	2000	81.3	1
Nigeria	Kebbi	42,654	Hypoendemic	2006	78.3	1
Nigeria	Kogi	1,952,921	Hyperendemic	2005	60.0	1
Nigeria	Kwara	1,100,580	Hyperendemic	2003	65.0	1
Nigeria	Niger	2,559,804	Mesoendemic	2004	78.6	1
Nigeria	Ogun	388,108	Mesoendemic	2003	81.9	1
Nigeria	Ondo	1,427,692	Mesoendemic	2007	65.0	1
Nigeria	Osun	1,813,101	Mesoendemic	2009	79.1	1
Nigeria	Oyo	907,621	Mesoendemic	2011	79.6	1
Nigeria	P20Nigeria	218,111	Hyperendemic	2015	79.7	1
Nigeria	P5Nigeria	3,206,285	Hypoendemic	No Tx	-	-
Nigeria	Plateau Nassarawa	799,473	Hyperendemic	2000	81.5	1
Nigeria	Taraba	733,815	Hyperendemic	2003	65.0	1
Nigeria	Yobe	161,626	Mesoendemic	2002	77.6	1
South Sudan	East Bahr El Ghazal	492,578	Hyperendemic	2011	70.9	1
South Sudan	East Equatoria	1,049,802	Hyperendemic	2009	66.9	1
South Sudan	P20SouthSudan	28,712	Hyperendemic	2015	65.0	1
South Sudan	Upper Nile	199,040	Hyperendemic	2010	65.8	1
South Sudan	West Bahr El Ghazal	1,429,133	Hyperendemic	2011	64.3	1

South Sudan	West Equatoria	690,536	Hyperendemic	2009	71.2	1
Sudan	P5Sudan	219,485	Hypoendemic	No Tx	-	-
Sudan	Sudan	50,904	Hypoendemic	2008	81.5	1
Uganda	Phase 1	57,989	Hyperendemic	2001	80.0	1
Uganda	Phase 2	82,506	Hypoendemic	2000	80.3	1
Uganda	Phase 3	739,243	Hyperendemic	2003	70.5	1
Uganda	Phase 4	101,196	Hyperendemic	2001	79.2	1
Uganda	Phase 5	234,044	Hyperendemic	2012	75.0	2

Notes: * Since 2013, skin biopsy on individuals of ≥5 years was performed in Burundi, Chad and Equatorial Guinea (mainland). [1] 18/20 surveyed villages in Burundi were *O. volvulus* mf-negative by skin snip, and two villages had low *O. volvulus* mf prevalence (<0.3%). All 23 surveyed villages in Chad were *O. volvulus* mf-negative by skin snip. 36/40 surveyed villages in Equatorial Guinea were *O. volvulus* mf-negative by skin snip, and four villages had low *O. volvulus* mf prevalence (<7.0%). For this reason, we assumed these areas to be no longer endemic for onchocerciasis.

[1] Pre-control endemicity levels as defined in Supplement S1, table S1.

[2] Year MDA initiation and population coverage originate from the MDA treatment database by the African Programme for Onchocerciasis Control (APOC) (up to November 2013), as previously published by Kim *et al.* [2] with some minor corrections. We followed the assumptions applied by Kim *et al.* concerning MDA treatment for hyper- and mesoendemic APOC projects that had not initiated MDA before 2013. Onchocerciasis hypoendemic areas would be excluded from MDA, as we assumed that alternative strategies would not yet have been implemented by 2025.

[3] Vector eliminated since 2005, and since no more cases infection have been reported. APOC project thus excluded from our analysis as no longer endemic for onchocerciasis [3].

References

1. WHO/APOC (2013) Remaining mapping challenges for onchocerciasis and *Loa*. Neglected Tropical Diseases Support Center. COR-NTD meeting. Washington DC. Accessed on: 13 February 2019. Available online at: https://www.ntdsupport.org/sites/default/files/uploads/docs/resources/14.%20%20Zoure%20-%20Remaining%20Oncho%20and%20loa%20loa%20mapping%20-%20revised.pdf.

2. Kim YE, Remme JHF, Steinmann P, Stolk WA, Roungou J-B, et al. (2015) Control, elimination, and eradication of river blindness: scenarios, timelines, and ivermectin treatment needs in Africa. *PLoS Negl Trop Dis* **9**: e0003664.

3. Herrador Z, Garcia B, Ncogo P, Perteguer MJ, Rubio JM, et al. (2018) Interruption of onchocerciasis transmission in Bioko Island: Accelerating the movement from control to elimination in Equatorial Guinea. *PLoS Negl Trop Dis* **12**: e0006471.
